# Supplementary material for: Mapping high resolution, multidimensional phase diagrams of near-physiological protein condensates
Source: Nat Commun. 2026 Sep 10;17:10711. doi: 10.1038/s41467-026-77696-6 (PMC13658236; doi:10.1038/s41467-026-77696-6)

Supplementary Information

Mapping high resolution, multidimensional phase diagrams of near-physiological protein condensates

Tanushree Agarwal[1], Tomas Sneideris[1], Fabian Svara[2], Klavs Jermakovs[1], Helena Coyle[3], Seema Qamar[3], Emanuel Kava[4], Rob Scrutton[1], Nicole Pleschka[3 †], Priyanka Peres[3], Gea Cereghetti[1], Ewa Andrzejewska[1], Alejandro Diaz-Barreiro[5,6], Gaby Palmer[5,6], Antonio J. Costa-Filho[4], Georg Krainer[1,7]*, Tuomas PJ Knowles[1]*, Jonathon Nixon-Abell[3]*

[1] Department of Chemistry, Centre for Misfolding Disease, University of Cambridge, Cambridge, UK

[2] ariadne.ai ag, Buchrain, Switzerland

[3] Cambridge Institute for Medical Research, Department of Clinical Neurosciences, University of Cambridge, Cambridge, UK

[4] Department of Physics, Ribeirão Preto School of Philosophy, Science and Literature, University of São Paulo and National Institute of Science and Technology in Innovative Research in Health Sciences: from Nanotechnology to Artificial Intelligence, Ribeirão Preto, Brazil

[5] Division of Rheumatology, Department of Medicine, Faculty of Medicine, University of Geneva, Geneva, Switzerland

[6] Department of Pathology and Immunology, Faculty of Medicine, University of Geneva, Geneva, Switzerland; Geneva Centre for Inflammation Research, Geneva, Switzerland

[7] Biophysics, Institute of Molecular Biosciences (IMB), University of Graz, NAWI Graz, Graz, Austria; Field of Excellence BioHealth, University of Graz, Graz, Austria; BioTechMed-Graz, Graz, Austria

[†] Current Address: Angiomed GmbH, Karlsruhe, Germany

* Corresponding authors: Jonathon Nixon-Abell (jjn36@cam.ac.uk), Tuomas PJ Knowles (tpjk2@cam.ac.uk), Georg Krainer (georg.krainer@uni-graz.at)

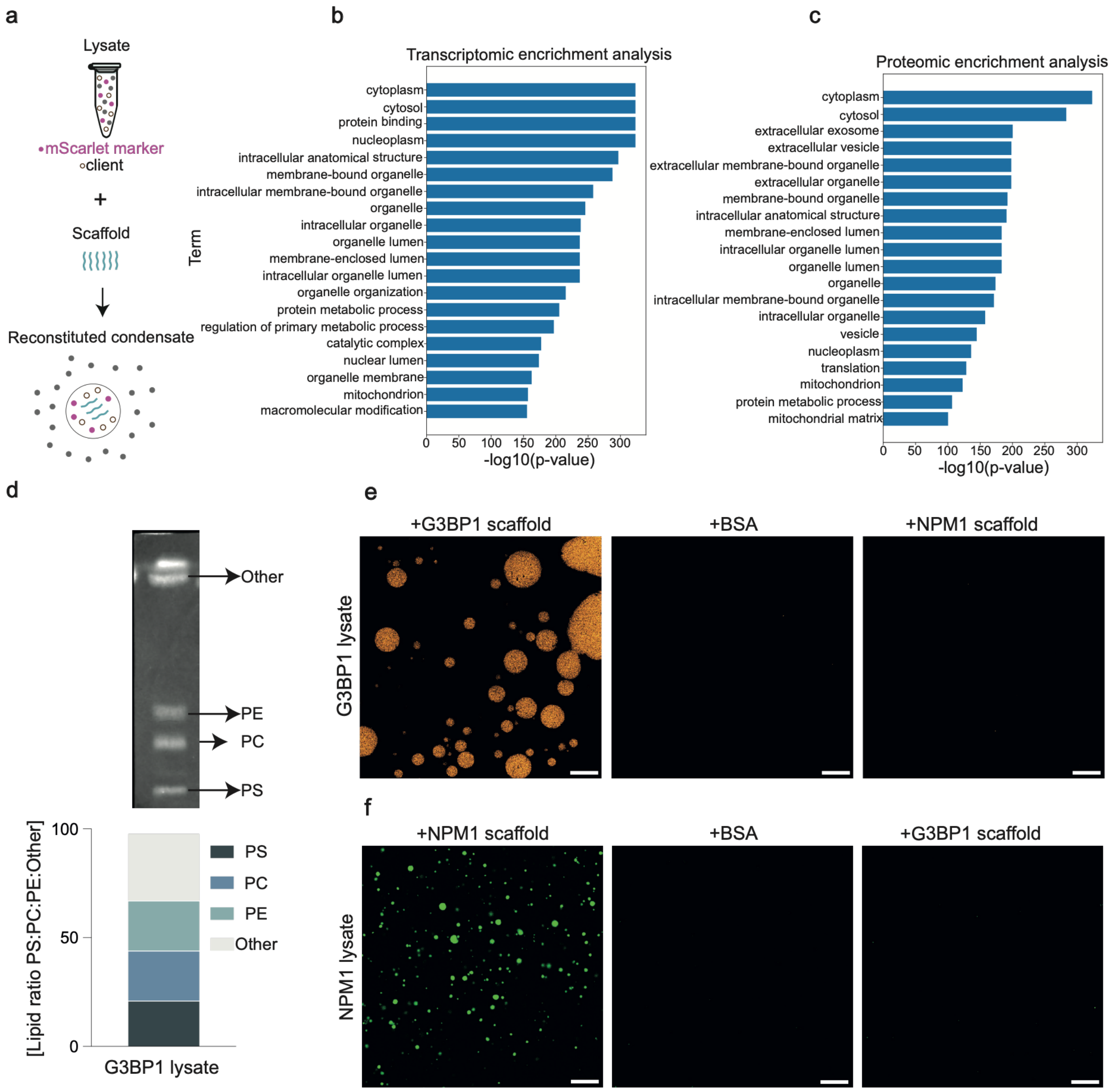

Supplementary Figure 1: Characterisation of lysate composition and specificity. **(a)** Schematic of lysate-based reconstitution where a recombinant scaffold nucleates condensates and recruits endogenous lysate components including mScarlet-tagged markers. **(b)** Transcriptomic enrichment analysis of G3BP1 lysate from Supplementary Table 1 (available in Source data). **(c)** Proteomic enrichment analysis of G3BP1 lysate from Supplementary Table 2 (available in Source data). **(d)** Thin Layer Chromotography (TLC) of G3BP1 lysate: gel-based separation (top) and stacked histogram of phosphatidylserine (PS), phosphatidylcholine (PC), phosphatidylethanolamine (PE) and other lipid classes (bottom). **(e-f)** Representative confocal images of lysate-reconstituted condensates demonstrating specificity: (e) Robust condensation with 1 mg/mL mScarlet-G3BP1 lysate and 15 µM G3BP1

scaffold (left), but no condensates with 15 μM BSA (centre) or 15 μM NPM1 scaffold (right). (f) Robust condensation with 4 mg/mL mScarlet-NPM1 lysate and 45 μM NPM1 scaffold (left), but not with 45 μM BSA (centre) or 45 μM G3BP1 scaffold (right). Scale bars, 10 μm.

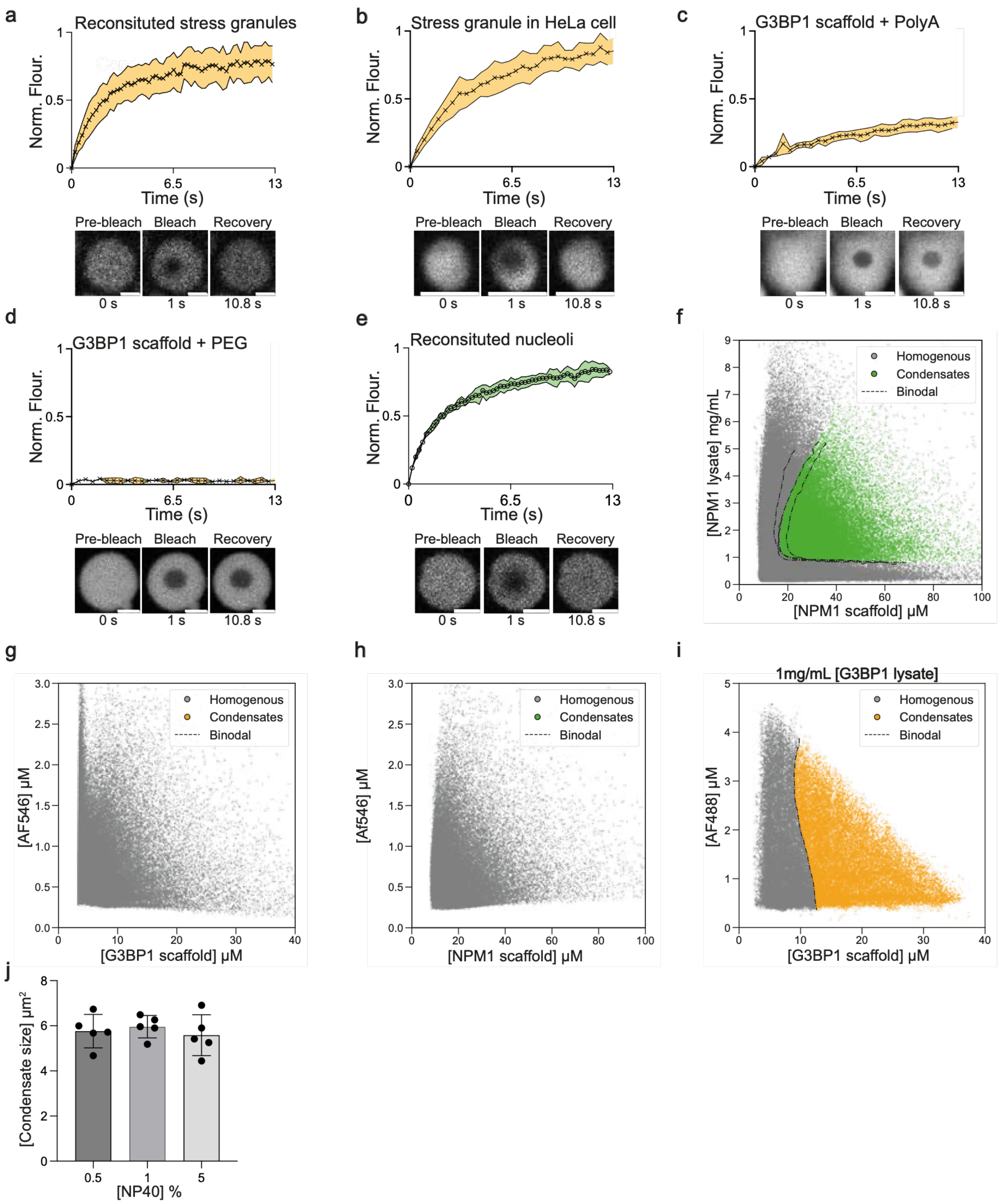

Supplementary Figure 2: Determining the efficacy of ExVivo PhaseScan (a-e) FRAP curves displaying fluorescence recovery (top) of reconstituted stress granules (15 μM G3BP1 scaffold + 1mg/mL G3BP1-mScarlet lysate) (a), stress granules in HeLa cells (HeLa stably expressing G3BP1-mScarlet) (b), 15 μM G3BP1 scaffold (AF647) + 10ng/μL Poly(A) RNA (c) and 15 μM G3BP1 scaffold (AF647) + 2% (w/v) PEG (10 kDa) (d), nucleoli (45 μM NPM1 scaffold + 4 mg/mL NPM1-mScarlet lysate) (e). Representative images (bottom) of pre-bleach, bleach and recovery timepoints are shown below. (a) n = 6, (b) n = 3, (c) n = 4, (d) n = 3, (e) n = 6 condensates; mean

± s.d. Scale bars, 3 µm. **(f)** Phase diagram of NPM1 scaffold versus NPM1-mScarlet lysate with phase boundaries from three independent experiments plotted as dashed lines, confirming reproducibility. Microdroplets are classified as homogeneous (grey) or condensate-containing (green). *n* = 466,222 microdroplets. **(g–h)** Phase diagram of AF647 labelled G3BP1 scaffold (g) or NPM1 scaffold (h) versus AF546 barcoded lysis buffer. Microdroplets classified as homogeneous (grey) or condensate-containing (G3BP1: yellow, NPM1: green). Note the lack of condensates. *n* = 125,528 (G3BP1); 53,693 (NPM1) microdroplets. **(i)** Phase diagram of G3BP1 scaffold at varying AF488 dye concentrations and constant 1 mg/mL G3BP1 lysate concentration. Microdroplets classified as homogeneous (grey) or condensate-containing (yellow). n = 64,595 microdroplets. **(j)** Quantification of the condensate size of reconstituted stress granules (15 µM G3BP1 scaffold + 1mg/mL G3BP1-mScarlet lysate) as a function of NP-40 concentration. Mean ± s.d., n = 5 independent fields of view (mean condensate size per FOV). One-way ANOVA with Tukey's multiple comparisons; ns ($P > 0.05$).

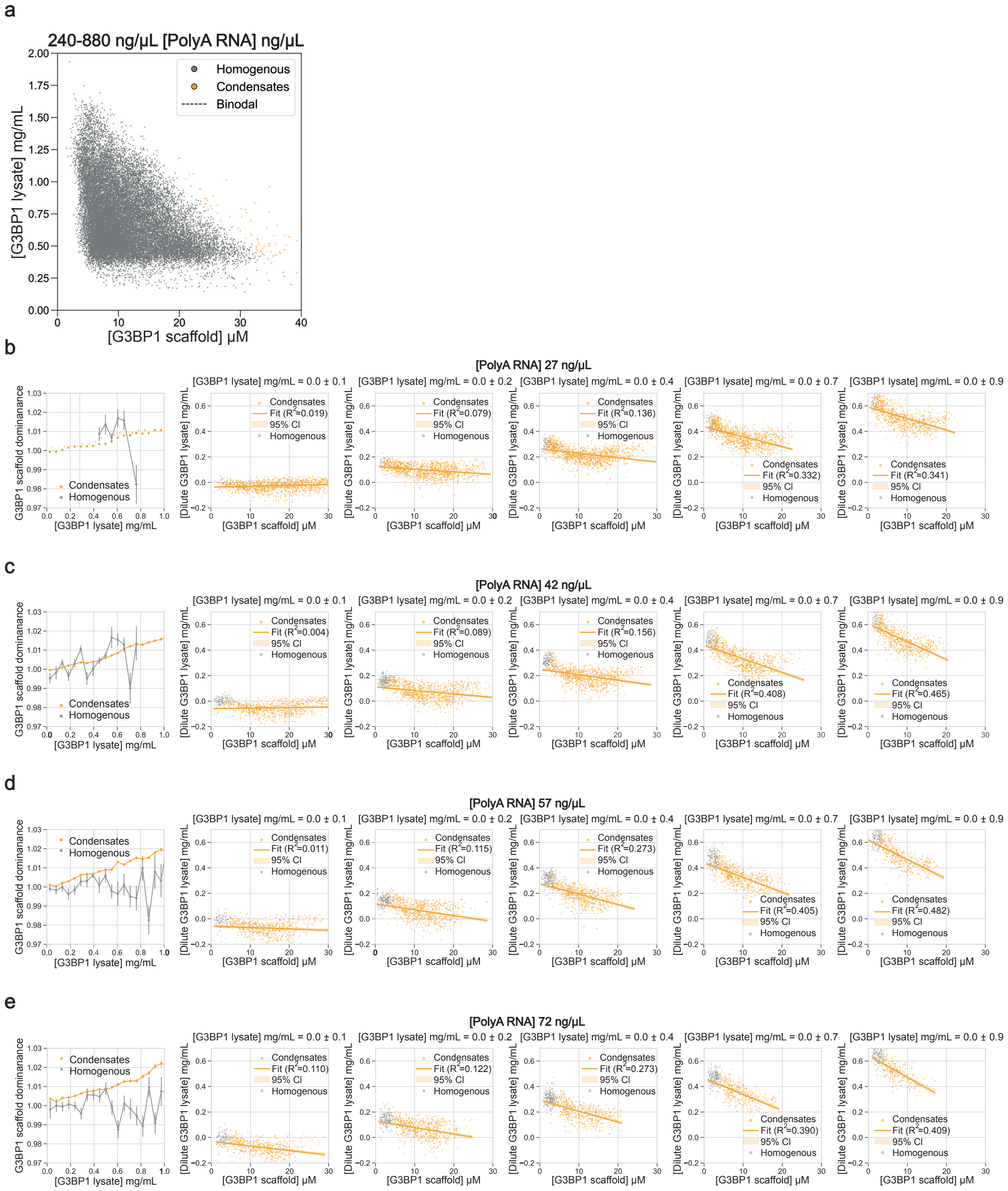

Supplementary Figure 3: Dominance framework analysis. (a) Phase diagram showing the effect of high AF488-barcoded Poly(A) RNA concentrations (240–880 ng/µL) on stress granule condensation. Each microdroplet is classed as homogeneous (grey) or condensate-containing (yellow). *n* = 28417 microdroplets. **(b–e)** Scatter plots showing the measured dilute phase mScarlet-G3BP1 lysate concentration (mg/mL) as a function of total recombinant G3BP1 scaffold concentration (µM) across increasing mScarlet-G3BP1 lysate concentrations (0.0–0.9 mg/mL) at four different Poly(A) RNA concentrations (27, 42, 57, and 72 ng/µL). Orange linear fits are shown with 95%

confidence intervals. The slope of each fit reflects the apparent dominance (D_{app}), prior to correction for unit mismatch (see Methods Section 12). Left most plots in each panel show the transition in G3BP1 scaffold apparent dominance across mScarlet-G3BP1 lysate concentration for each Poly(A) RNA level.

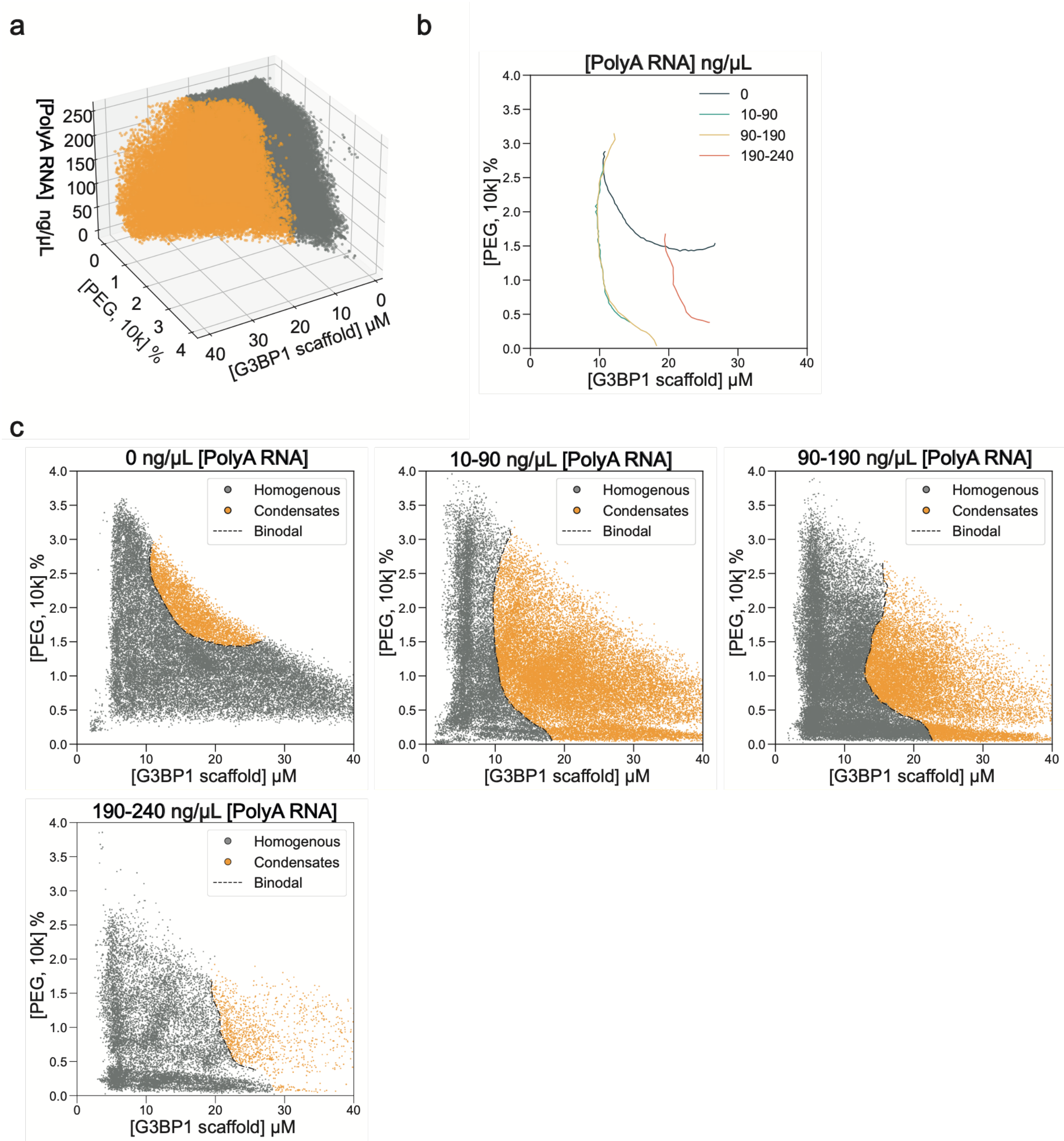

Supplementary Figure 4: Phase diagrams showing the effect of Poly(A) RNA on G3BP1 scaffold condensates with PEG, 10 KDa. (a-b) Three-dimensional phase diagram showing the effect of showing the effect of increasing AF546-barcoded Poly(A) RNA concentration on G3BP1-PEG, 10kDa condensation (a) and extracted phase boundaries (b). **(c)** Two-dimensional slices through the phase diagrams as displayed in (a) showing the effect of increasing AF546-barcoded Poly(A) RNA concentrations (0 ng/μL, 10–90 ng/μL, 90–190 ng/μL, 190–240 ng/μL) on G3BP1-PEG condensation. *n* = 32,783; 45,681; 55,329; 14,990 microdroplets. Microdroplets throughout are classified as homogeneous (grey) or condensate-containing (yellow), with the binodal phase boundary plotted as a dashed line.

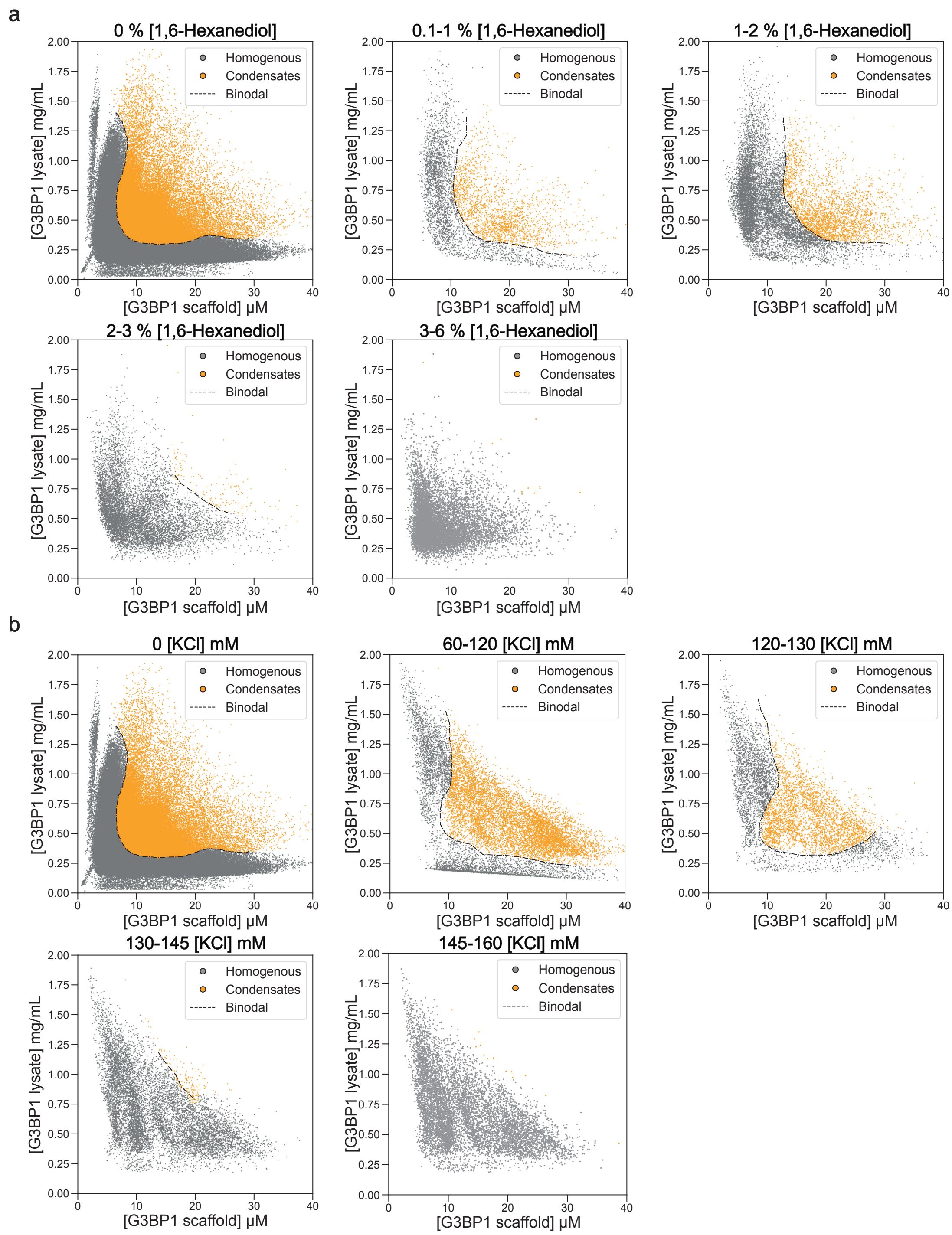

Supplementary Figure 5: Phase diagrams showing the effect of 1,6-Hexanediol and KCl on stress granule condensation. (a) Phase diagrams showing the effect of Increasing AF488-barcoded 1,6-Hexanediol concentrations (0%, 0.1–1%, 1–2%, 2–3%, 3–6%) on stress granule condensation. *n* = 155,584; 4,827; 11,461; 7,953; 9,361 microdroplets. **(b)** Phase diagrams showing the effect of Increasing AF488-barcoded KCl concentrations (0 mM, 60–120 mM, 120–130 mM, 130–145 mM, 145–160 mM) on stress granule condensation. *n* = 155,584; 14,314; 6,577; 9,413; 8,010

microdroplets, respectively. Microdroplets throughout are classified as homogeneous (grey) or condensate-containing (yellow), with the binodal phase boundary plotted as a dashed line.

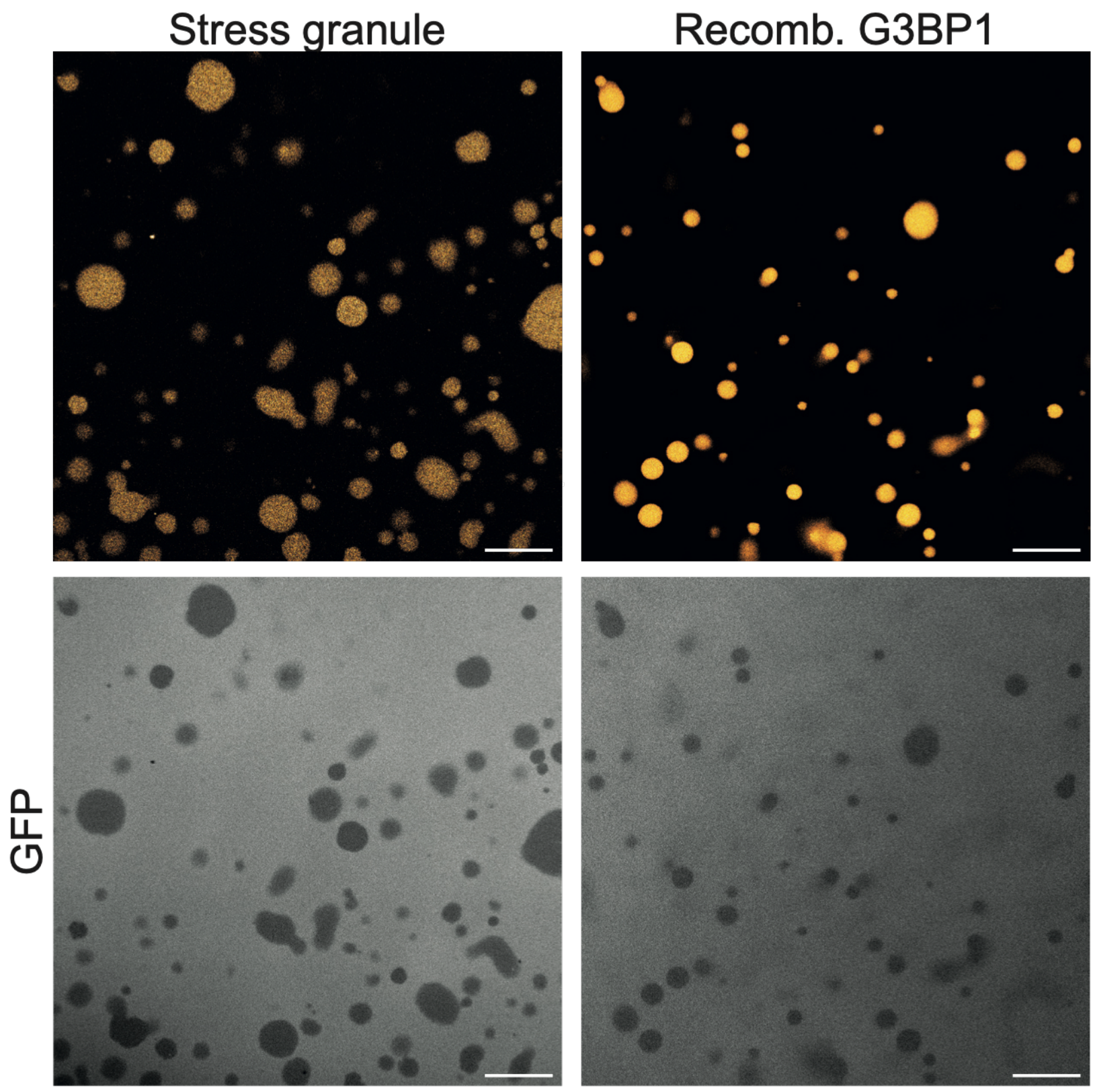

Supplementary Figure 6: Purified GFP does not localise to reconstituted stress granules or recombinant-only G3BP1 condensates. Confocal images of lysate-reconstituted stress granules (15 µM G3BP1 + 1 mg/mL mScarlet-G3BP1 lysate) and recombinant-only G3BP1 condensates (15 µM AF647-G3BP1 + 10 ng/µL PolyA RNA) in the top row. Bottom row shows that 1 µM recombinant GFP is not incorporated into either condensate. Scale bars, 20 µm.

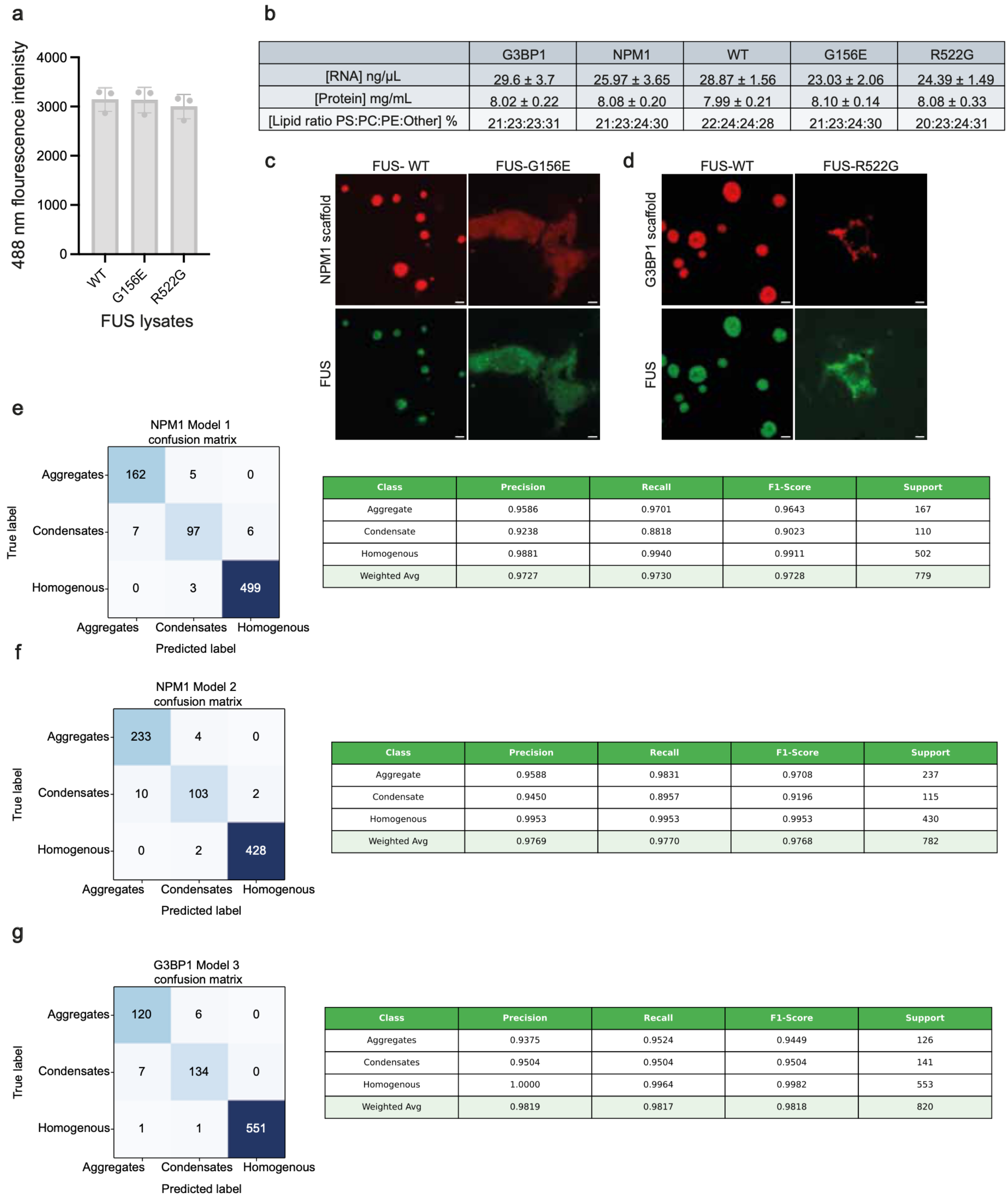

	G3BP1	NPM1	WT	G156E	R522G
[RNA] ng/µL	29.6 ± 3.7	25.97 ± 3.65	28.87 ± 1.56	23.03 ± 2.06	24.39 ± 1.49
[Protein] mg/mL	8.02 ± 0.22	8.08 ± 0.20	7.99 ± 0.21	8.10 ± 0.14	8.08 ± 0.33
[Lipid ratio PS:PC:PE:Other] %	21:23:23:31	21:23:24:30	22:24:24:28	21:23:24:30	20:23:24:31

Class	Precision	Recall	F1-Score	Support
Aggregate	0.9586	0.9701	0.9643	167
Condensate	0.9238	0.8818	0.9023	110
Homogenous	0.9881	0.9940	0.9911	502
Weighted Avg	0.9727	0.9730	0.9728	779

Class	Precision	Recall	F1-Score	Support
Aggregate	0.9588	0.9831	0.9708	237
Condensate	0.9450	0.8957	0.9196	115
Homogenous	0.9953	0.9953	0.9953	430
Weighted Avg	0.9769	0.9770	0.9768	782

Class	Precision	Recall	F1-Score	Support
Aggregates	0.9375	0.9524	0.9449	126
Condensates	0.9504	0.9504	0.9504	141
Homogenous	1.0000	0.9964	0.9982	553
Weighted Avg	0.9819	0.9817	0.9818	820

Supplementary Figure 7: Machine learning classification of FUS condensate morphology. (a) Quantification of 488nm fluorescence intensity of prepared mEmerald-FUS lysates (WT, G156E, R522G). Mean ± s.d., n = 3 independent lysate samples. One-way ANOVA with Tukey's multiple comparisons; ns ($P > 0.05$) for all groups. **(b)** Quantification of RNA and protein concentration, and lipid composition across lysates (G3BP1-mScarlet, NPM1-mScarlet and FUS-mEmerald WT, G156E and R522G), RNA and protein values are mean ± s.d., n = 3 independent lysate samples. **(c)** Confocal images of reconstituted nucleoli formed using 45 µM AF647-

labelled NPM1 scaffold with 4 mg/mL mEmerald-FUS WT lysate (left) or G156E lysate (right). **(d)** Confocal images of reconstituted stress granules formed using 12.5 µM AF647-labelled G3BP1 scaffold with 5 mg/mL mEmerald-FUS WT lysate (left) or R522G lysate (right). (c-d) Scaffold channel (top) and FUS channel (bottom). Scale bars, 2 µm. **(e-g)** Confusion matrices and corresponding performance metrics (precision, recall, F1-score, and support) shown for three classification models used to distinguish between homogenous, condensate, and aggregate droplet morphologies. Metrics calculated on held-out test set comprising 5% of respective dataset. Weighted average scores summarize the overall performance across all classes, with each class score weighted by the number of class true instances.

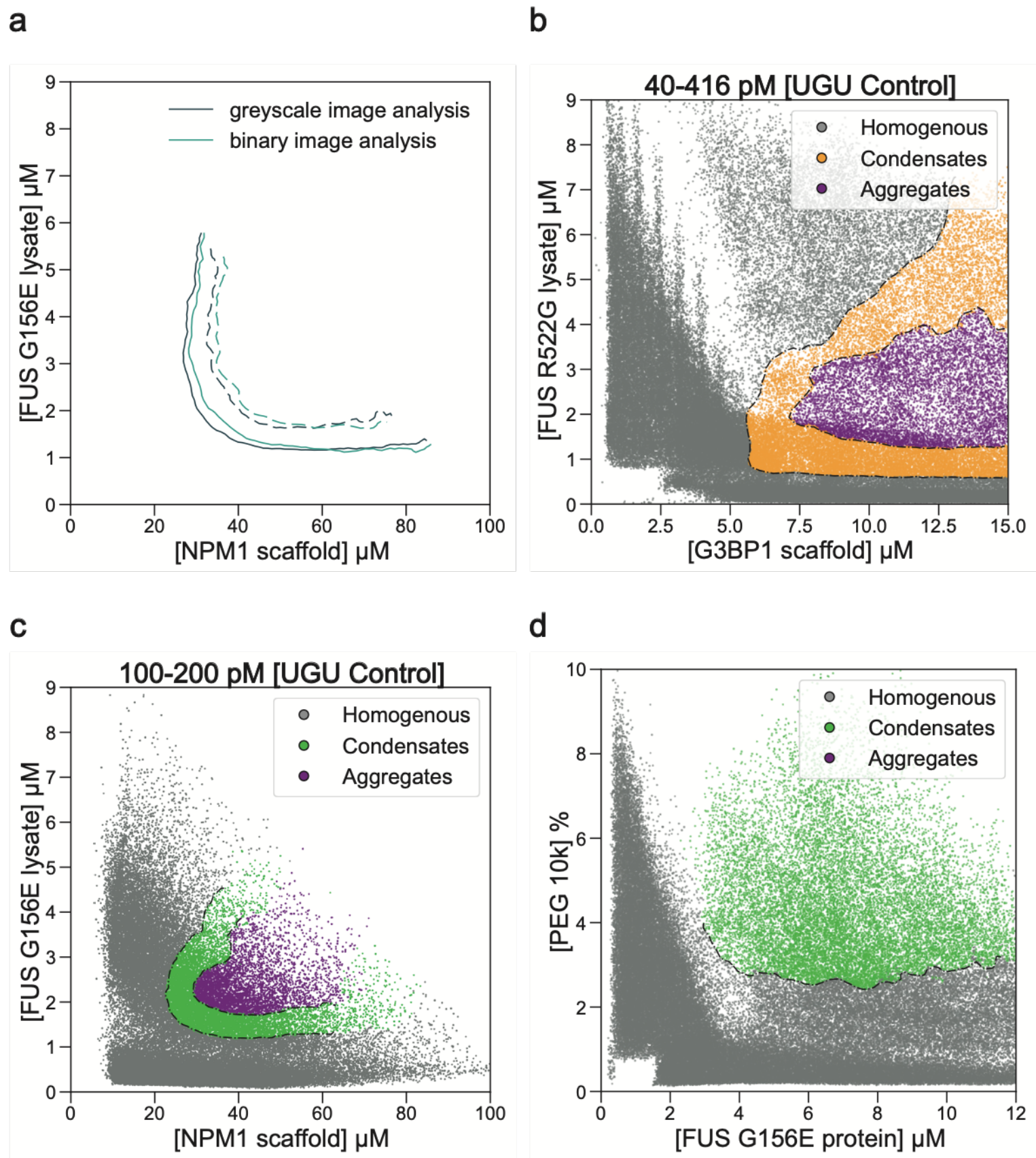

Supplementary Figure 8: RNA aptamer-based modulation of FUS phase behaviour in stress granules. (a) Boundary extraction from grayscale and binary image analysis (see Supplementary Text, Section 2.2). Solid line: condensate boundary**,** dashed line: aggregation boundary **(b–c)** Phase diagrams demonstrating the effect of a AF546-barcoded scrambled UGU control aptamer on mEmerald-FUS R522G containing stress granules (b), and mEmerald-FUS G156E containing nucleoli (c). n= 109,195 (R522G); 81,601(G165E) microdroplets. **(d)** Phase diagram of purified G156E-GFP versus PEG 10k. *n* = 90,108; Microdroplets are classified throughout as homogeneous (grey), condensate-containing (green/yellow), or aggregated (purple), with the binodal phase boundaries plotted as a dashed line.

Supplementary Text

1.1 Dominance analysis framework

To extract the dominance from our reconstituted stress granule system, we applied the method previously outlined in Qian et al., 2024 [1] This consisted, for each individual microdroplet, calculating the total concentration and the dilute phase concentration, from the mean and 5-25% pixel intensity values respectively. Then, our 3D phase diagram was sliced into volumes of 15ng/uL RNA. In each volume, we plot each droplet's recombinant G3BP1 concentration against the dilute fluorescently tagged G3BP1-lysate concentration. The gradient of this plot for the phase separated droplets gives a response (R) related to the dominance via D = 1-R. As dominance is a dimensionless quantity, we apply a unit correction outlined in Methods Section 12. Combining the changes in dominance as a function of component concentration, with changes in the saturation concentration, we relate our observations to one of the 4 scenarios outlined in Qian et al., 2024 [1]: Target/Auxilliary Enhancement/Suppression.

2.1 Machine learning-based microdroplet classifier

To enable scalable and unbiased analysis in ExVivo PhaseScan, we developed an automated machine learning pipeline to classify individual microdroplet images as homogeneous*,* condensate*,* or aggregate. Given the throughput of ExVivo PhaseScan, we decided to use morphology-based features as a scalable readout of condensate state within each droplet. Training labels were assigned based on morphology, using criteria informed by previously reported quantitative descriptors of condensate-to-aggregate transitions, including intensity, area, roundness, circularity and aspect ratio [4]. Homogeneous droplets were defined as droplets with diffuse signal and no internal puncta. Condensate-containing droplets were defined by the presence of rounded, droplet-like internal structures. Aggregate-containing droplets were defined by irregular, high-intensity, non-spherical structures with reduced circularity and more heterogeneous internal morphology. The number of labelled examples used for training were as follows: NPM1 Model 1: 3,824 aggregates, 2,481 condensates, and 9,257 homogenous. NPM1 Model 2: 3,860 aggregates, 2,524 condensates, and 9,257 homogenous. G3BP1 Model 3: 2,748 aggregates, 2,725 condensates, and 10,924 homogenous. Individual droplet images were first segmented, cropped, and processed through a pre-trained MobileNetV2 convolutional

neural network [2] implemented via TorchVision [3]. From the final layer, we extracted 1280-dimensional feature embeddings encoding high-level spatial and textural information of the droplets. These embeddings were then used as input to an XGBoost classifier trained to distinguish three distinct morphological classes: (i) Class 1 (Homogeneous): even intensity throughout the droplet with no visible internal features; (ii) Class 2 (Condensates): presence of small in ternal puncta or dot-like patterns, indicative of liquid-like condensation; (iii) Class 3 (Aggregates): irregular internal structures consistent with misfolded or aggregated material.

2.2 Microdroplet classifier validation

To demonstrate that our classification pipeline relies on condensate morphological features instead of exploiting obscure class-specific pixel intensity patterns, we compared boundary detection using per droplet normalised grayscale images (0–255 pixel values) and binarised image masks (converted to 0–1). We converted grayscale images to binary masks with a pixel value of 1 assigned to objects (condensates/aggregates) and 0 to the remaining image. Boundaries extracted from both intensity-based and binarised masks approaches are extremely similar (Supplementary Figure 8a), ensuring that our analysis relies on morphology-based features rather than class-specific information leakage. These results highlight the robustness of the pipeline in resolving subtle morphological differences and demonstrate its broad utility for high-throughput droplet phenotyping.

References

1. Qian, D., et al., *Dominance analysis to assess solute contributions to multicomponent phase equilibria.* Proc Natl Acad Sci U S A, 2024. **121**(33): p. e2407453121
2. Sandler, M., et al., *MobileNetV2: Inverted Residuals and Linear Bottlenecks*. Proc IEEE Conf Comput Vis Pattern Recognit, 2018. 4510-4520.
3. (https://pytorch.org/vision/stable/index.html)
4. Silva, I. R. E., Dariani, G. G., Benevenutti, F. Z., de Oliveira, R. R., Nicoleti, V. Y. U. & de Carvalho, M. *A computational pipeline for image-based statistical analysis of biomolecular condensates dynamics using morphological descriptors*. Sci. Rep. **15**, 27560 (2025).

Uncropped gel from Supplementary Figure 1d

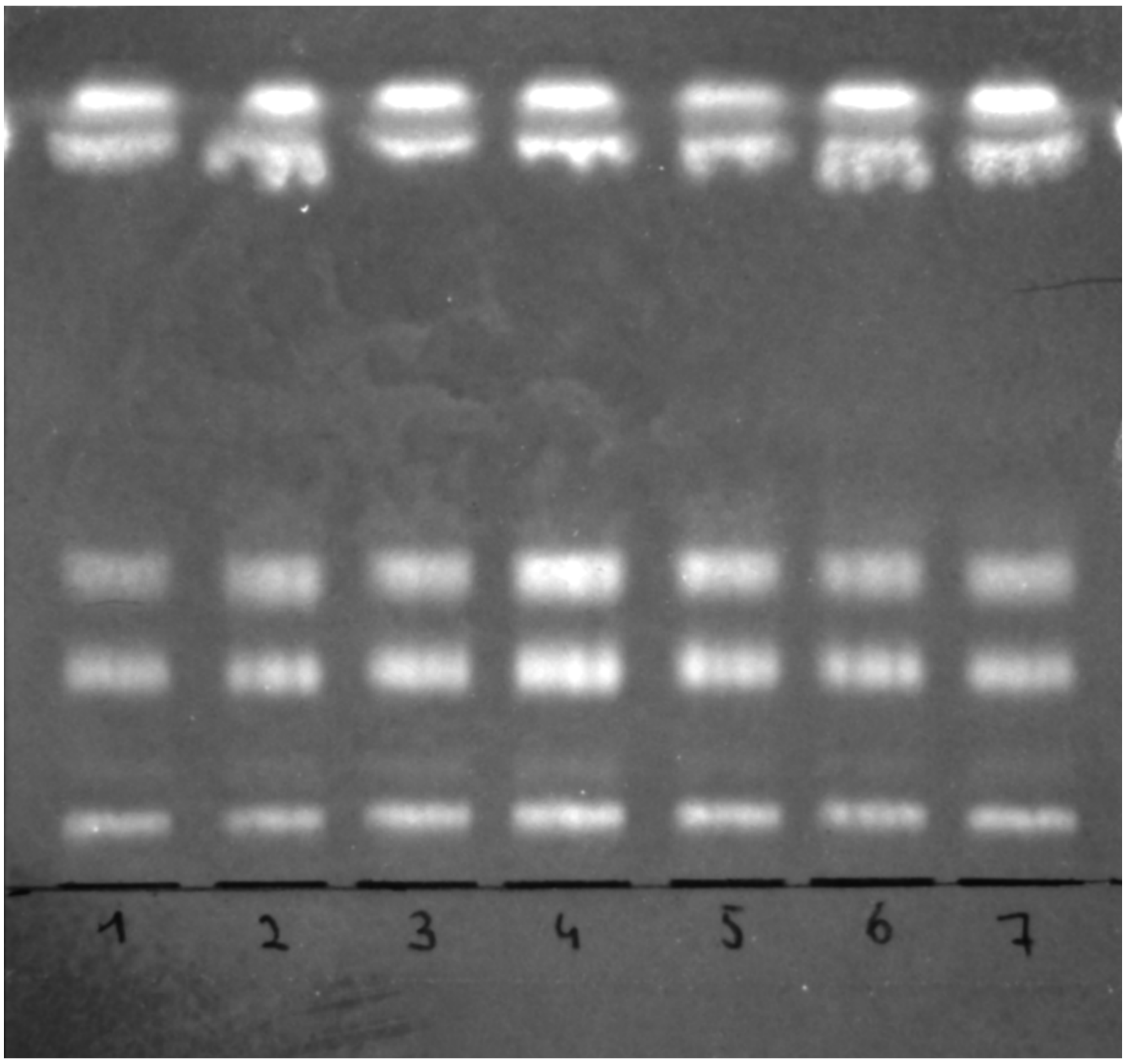

Supplement: Supplementary file 1 [file 41467_2026_77696_MOESM1_ESM.pdf]
