# Supplementary material for: Mapping high resolution, multidimensional phase diagrams of near-physiological protein condensates
Source: Nat Commun. 2026 Sep 10;17:10711. doi: 10.1038/s41467-026-77696-6 (PMC13658236; doi:10.1038/s41467-026-77696-6)
Supplement: Supplementary file 2 [file 41467_2026_77696_MOESM2_ESM.pdf]

nature portfolio

Corresponding author(s): TGK, TPJK, JNA

Last updated by author(s): 05/09/2026

Reporting Summary

Nature Portfolio wishes to improve the reproducibility of the work that we publish. This form provides structure for consistency and transparency in reporting. For further information on Nature Portfolio policies, see our Editorial Policies and the Editorial Policy Checklist.

Statistics

For all statistical analyses, confirm that the following items are present in the figure legend, table legend, main text, or Methods section.

n/a	Confirmed	
☐	☒	The exact sample size (*n*) for each experimental group/condition, given as a discrete number and unit of measurement
☐	☒	A statement on whether measurements were taken from distinct samples or whether the same sample was measured repeatedly
☐	☒	The statistical test(s) used AND whether they are one- or two-sided<br>*Only common tests should be described solely by name; describe more complex techniques in the Methods section.*
☐	☒	A description of all covariates tested
☐	☒	A description of any assumptions or corrections, such as tests of normality and adjustment for multiple comparisons
☐	☒	A full description of the statistical parameters including central tendency (e.g. means) or other basic estimates (e.g. regression coefficient) AND variation (e.g. standard deviation) or associated estimates of uncertainty (e.g. confidence intervals)
☐	☒	For null hypothesis testing, the test statistic (e.g. *F*, *t*, *r*) with confidence intervals, effect sizes, degrees of freedom and *P* value noted<br>*Give P values as exact values whenever suitable.*
☒	☐	For Bayesian analysis, information on the choice of priors and Markov chain Monte Carlo settings
☒	☐	For hierarchical and complex designs, identification of the appropriate level for tests and full reporting of outcomes
☒	☐	Estimates of effect sizes (e.g. Cohen's *d*, Pearson's *r*), indicating how they were calculated

Our web collection on statistics for biologists contains articles on many of the points above.

Software and code

Policy information about availability of computer code

Data collection	ZEN Black v2.3 (Zeiss), Micro-Manager version 2.0, NovaSeq X Series Control Software, Illumina bcl2fastq program version 2.20, DIA-NN v2.2
Data analysis	FIJI v1.54d (NIH), PRISM 9 v10.4.1(Graphpad), AutoCAD 2025, Adobe Illustrator v30.7, Python v3.9, g:Profiler 0.3.5, Trimmomatic v.0.36, STAR aligner v.2.5.2b, Subread package v.1.5.2, encyclopeDIA (v2.12.30). All code used in this study is available at GitHub, for PhaseScan analysis (https://github.com/rqi14/PhaseScan), Machine Learning-assisted droplet classification and morphology-based analysis (https://github.com/kjermakovs/DropletClassification) ,and dominance analysis (https://github.com/Rob-Scrutton/DominanceAnalysis).

For manuscripts utilizing custom algorithms or software that are central to the research but not yet described in published literature, software must be made available to editors and reviewers. We strongly encourage code deposition in a community repository (e.g. GitHub). See the Nature Portfolio guidelines for submitting code & software for further information.

Data

Policy information about availability of data

All manuscripts must include a data availability statement. This statement should provide the following information, where applicable:

- Accession codes, unique identifiers, or web links for publicly available datasets
- A description of any restrictions on data availability
- For clinical datasets or third party data, please ensure that the statement adheres to our policy

All data analysed during this study are included in this published article and its supplementary information files. Source data are provided with this paper. Raw data files are available on Figshare under accession code 10.6084/m9.figshare.30630299. RNA-seq data were deposited into ArrayExpress/BioStudies under accession

number E-MTAB-17121 and are available at the following URL: https://www.ebi.ac.uk/biostudies/arrayexpress/studies/E-MTAB-17121. Mass spectrometry proteomics data were deposited into the ProteomeXchange Consortium via the PRIDE partner repository under accession number PXD079312 and are available at the following URL: https://www.ebi.ac.uk/pride/archive/projects/PXD079312. Graphs were plotted using GraphPad Prism (v10.4.1) and schematic figure panels were prepared using Adobe Illustrator (v30.7).

Research involving human participants, their data, or biological material

Policy information about studies with human participants or human data. See also policy information about sex, gender (identity/presentation), and sexual orientation and race, ethnicity and racism.

Reporting on sex and gender	N/A
Reporting on race, ethnicity, or other socially relevant groupings	N/A
Population characteristics	N/A
Recruitment	N/A
Ethics oversight	N/A

Note that full information on the approval of the study protocol must also be provided in the manuscript.

Field-specific reporting

Please select the one below that is the best fit for your research. If you are not sure, read the appropriate sections before making your selection.

[x] Life sciences [ ] Behavioural & social sciences [ ] Ecological, evolutionary & environmental sciences

For a reference copy of the document with all sections, see nature.com/documents/nr-reporting-summary-flat.pdf

Life sciences study design

All studies must disclose on these points even when the disclosure is negative.

Sample size	Sample sizes for experiments are clearly stated throughout the manuscript. The n number of experiments is listed alongside the total number of points analysed per experiment. Sample sizes were selected based on variance observed in prior experiments of a similar nature and to meet appropriate requirements to permit statistical interrogation of the data.
Data exclusions	No data were excluded from the analyses
Replication	All replicates are included in the manuscript, all attempts at replication were successful
Randomization	No formal randomization method was required for these types of data.
Blinding	Investigators were not blinded to the experimental group allocation as the analysis methods employed within this study are automated

Reporting for specific materials, systems and methods

We require information from authors about some types of materials, experimental systems and methods used in many studies. Here, indicate whether each material, system or method listed is relevant to your study. If you are not sure if a list item applies to your research, read the appropriate section before selecting a response.

Materials & experimental systems

n/a	Involved in the study	
[x]	[ ]	Antibodies
[ ]	[x]	Eukaryotic cell lines
[x]	[ ]	Palaeontology and archaeology
[x]	[ ]	Animals and other organisms
[x]	[ ]	Clinical data
[x]	[ ]	Dual use research of concern
[x]	[ ]	Plants

Methods

n/a	Involved in the study	
[x]	[ ]	ChIP-seq
[x]	[ ]	Flow cytometry
[x]	[ ]	MRI-based neuroimaging

Eukaryotic cell lines

Policy information about cell lines and Sex and Gender in Research

Cell line source(s)	HeLa cells (ATCC), E. coli Rosetta (DE3) cells (Sigma) , E. coli BL21 (DE3) (NEB)
Authentication	Authentication details are listed on the ATCC, Sigma, NEB webpage
Mycoplasma contamination	Cell lines were tested for mycoplasma by PCR on a monthly basis. All lines tested negative
Commonly misidentified lines (See ICLAC register)	N/A
