# Supplementary material for: Mapping high resolution, multidimensional phase diagrams of near-physiological protein condensates
Source: Nat Commun. 2026 Sep 10;17:10711. doi: 10.1038/s41467-026-77696-6 (PMC13658236; doi:10.1038/s41467-026-77696-6)
Supplement: Supplementary file 3 [file 41467_2026_77696_MOESM3_ESM.pdf]

nature portfolio

Peer Review File

Mapping high resolution, multidimensional phase diagrams of near-physiological protein condensates

Corresponding Author: Dr Jonathon Nixon-Abell

This file contains all reviewer reports in order by version, followed by all author rebuttals in order by version.

Version 0:

Reviewer comments:

Reviewer #1

(Remarks to the Author)
This manuscript presents a nice approach by using cell lysate as a biologically relevant crowding environment to study liquid-liquid phase separation (LLPS), moving beyond the use of synthetic crowders such as PEG/dextran in the in vitro studies. The overall concept is well motivated and has the potential to significantly advance the field by bringing in vitro LLPS studies closer to physiological conditions. However, in its current form, the manuscript requires several improvements to strengthen mechanistic insight, methodological clarity, and contextualization. I therefore recommend major revision before the work can be considered for publication.
1. The central novelty of the study lies in the use of cell lysate, which inherently contains a complex mixture of proteins, lipids, nucleic acids, and other biomolecules. Despite this, if I understand correctly, the lysate concentration is largely treated as equivalent to protein concentration throughout the manuscript. While proteins may represent the dominant component, other biomolecules similar to the crowders used in the in vitro systems are known to play important roles in LLPS and may significantly influence the observed phase behaviour. This simplifying assumption limits mechanistic interpretation and underutilizes the key strength of the experimental design.
2. A more systematic characterization of the lysate composition is therefore necessary. At present, the absence of detailed compositional analysis makes it difficult to assess the relative contributions of different biomolecular classes. Even a coarse or semiquantitative analysis of major components, at minimum proteins, nucleic acids, lipids, and relevant inorganic salts, would provide essential context and greatly improve the depth and rigor of the study. This is important as in different physiological conditions, these molecules may present a different concentration profile.
3. The rationale behind the concentration dependence of the lysate also requires clearer explanation. The authors should explicitly discuss what physicochemical changes are expected as lysate concentration is varied and why protein concentration alone is used as the defining parameter. Clarifying this point would significantly improve transparency in experimental design and data interpretation. In this context, it would also strengthen the manuscript to include a discussion of potential differences between lysates derived from normal versus diseased cells. While experimental validation may be challenging, even a conceptual or simulation based discussion of disease associated changes in lysate composition and their possible consequences for LLPS would broaden the relevance of the work.
4. The manuscript does not compare with existing in vitro LLPS studies involving the proteins of interest, particularly those conducted using defined crowding agents. Such a comparison is important to clearly understand what new insights are placed forward by the lysate based approach and to properly position the study within the existing LLPS literature.
5. Several methodological details require clarification. In particular, the preparation of recombinant only condensates is insufficiently described, limiting reproducibility. Additionally, Figure 1a shows recombinant only condensates with predominantly circular morphology, whereas Figure 3b shows both recombinant only and lysate based condensates with irregular shapes. The authors should clarify whether this discrepancy arises from differences in experimental conditions, imaging parameters, or underlying material properties.
6. To substantiate the claim that lysate based condensates more closely resemble cellular condensates, direct and quantitative comparisons are needed. Specifically, material properties of cellular, lysate based, and recombinant only condensates may be compared using FRAP analysis. Without such comparisons, the claims are qualitative.
7. The interpretation of client partitioning data in Figure 3b requires some attention.
First, client partitioning depends on both condensate material properties and scaffold client interactions. When the scaffold and nominal phase appear similar, what accounts for the differential partitioning of clients such as FUS, IL38, and PKM2? If the material phases of lysate based and recombinant only condensates differ, this should be clearly demonstrated and discussed before comparing partitioning behaviour using the same client molecules. Next, the lower fluorescence intensity

observed for IL38 relative to FUS or PKM2 within condensates also requires explanation.
8. Also, the protein expression conditions raise concerns. The use of 500 mM IPTG for IL38 overexpression in E. coli BL21 (DE3) cells is unusually high and may promote protein aggregation. Is it a typo? The authors should clarify this choice and provide justification or evidence supporting proper protein folding and functionality under these conditions.
The experimental concept is strong and more towards physiology of course, however, the current form of the manuscript does not yet justifies that. Addressing the above points would enhance the clarity and impact of the work and the conclusions of the study.

(Remarks on code availability)

Reviewer #2

(Remarks to the Author)
Tanushree and colleagues present a lysate-based microfluidic platform aiming at generating high resolution phase diagram especially for physiological protein condensates. The platform, named ExVivo PhaseScan, was identical to their 2022 nature comm paper (called PhaseScan back then). The only difference I can tell is that they replace the in-vitro medium with cell lysates to mimic in-vivo conditions. So I think that technical advancement of this manuscript is minor. Application-wise, they described the lysate-reconstitution and applied the platform to dissect stress granule assembly and provide pathological insights. However, further evidence and much greater experimental rigor is needed to substantiate their claims and provide a truly novel advance in this field. Below are my concerns and comments
Major:
1. Fig2: why not also barcoding concentrations of RNA/1,6-HEXANEDIOL/KCl using an additional dye color? In this way the screening would be in higher dimension and more comprehensive. It seems (in panel b) that these additives are in a broad range of concentrations rather than specific values. What’s the point of doing that? did these concentrations also undergo arbitrary dilution through microfluidics? If so, I highly recommend barcode them as well. It is weird to put the data got from different RNA/1,6-HEXANEDIOL/KCl concentrations in the same plot. Or why not just do a serial dilution set of RNA/1,6-HEXANEDIOL/KCl concentrations instead of giving a range? It is at least clearer to see the differences if any.
2. Lines 137–139: The claim that lysate-derived components are essential for condensation conflicts with the cannonical scaffold/client framework. Please clarify the specific role of these components in more detail.
3. A key strength of this study is the use of lysates to introduce presumably physiological, endogenous components and native cofactors. However, the lysates were derived from cells overexpressing G3BP1/NPM1 and prepared with NP40 (containing detergent) and RNase inhibitors. Furthermore, in the validation of client recruitment, the client proteins were also exogenously overexpressed, and precise modulation of biochemical variables was achieved by artificially adding exogenous mimics. Additionally, the identity and concentration of the native components retained in the lysates that could be recruited into the condensates were neither mentioned nor investigated.
4. The authors used the ExVivo PhaseScan platform to study stress granule assembly, variable modulation, and condensate maturation. While the observations align with the cited literature, novel findings that specifically demonstrate the platform's advantages over PhaseScan would better highlight its significance.
Minor:
1. Fig1: I’m wondering whether the addition of fluorescent dye molecules affects the phase separation behavior?
2. Abstract: it would be better to identify ALS and FUS at the very first place of using these abbreviations.
3. Several claims of "for the first time" in the Discussion appear overstated based on the data presented.

(Remarks on code availability)

Reviewer #3

(Remarks to the Author)

Agarwal et al. present ExVivo PhaseScan, an innovative droplet-based microfluidic platform designed to bridge the gap between the biochemical complexity of cellular lysates and the high-throughput, parameter-controlled precision of in vitro microfluidics. Using this approach, the authors generate high-resolution phase diagrams for two major biomolecular condensates—stress granules and nucleoli—and systematically examine the roles of RNA concentration, hydrophobic interactions, and electrostatic forces in driving phase separation. A major strength of the study is the demonstration that this lysate-based system more faithfully recapitulates client protein recruitment than simplified recombinant reconstitution assays. In addition, the integration of a machine-learning–based image classifier enables quantitative analysis of condensate morphology, revealing that ALS-associated FUS mutations shift the phase landscape toward pathological solid or aggregated states. Importantly, the authors further show that this aberrant behavior can be partially reversed using a specific RNA aptamer.
Overall, the data are of high quality, and the resulting phase diagrams exhibit impressive resolution. The application of machine learning for automated morphological classification represents a notable methodological advance. However, despite these strengths, I do not consider the manuscript to meet the conceptual or mechanistic threshold for Nature Communications. More detailed concerns are outlined below.

Major points:

1. Quantitative characterization of phase diagrams for multicomponent biomolecular condensates under near-physiological conditions is essential for elucidating the molecular mechanisms governing condensate composition and regulation. However, conventional approaches face inherent limitations: in vitro reconstitution systems are often overly simplified, whereas in vivo cellular assays are difficult to control and systematically manipulate. To address this gap, Agarwal et al. introduce a microfluidic strategy in which cell lysates are incubated with scaffold proteins, enabling high-throughput sample preparation and real-time visualization of condensate formation. This approach allows rapid construction of quantitative phase diagrams for stress granules and nucleoli.
Despite these technical advances, the conceptual distinction between the present study and the authors' 2022 work (https://www.nature.com/ articles/s41467-022-35265-7), appears limited, aside from replacing a defined component with cell lysate. While incorporating lysates is a reasonable attempt to enhance physiological relevance and potentially extend the PhaseScan platform toward drug-screening applications—an advance of clear interest to the biophysics, cell biology, and pharmacology communities—it raises a fundamental concern regarding how faithfully cell lysates recapitulate intracellular conditions.
First, lysate preparation requires substantial buffer dilution and includes NP40, a detergent that may perturb intermolecular interactions critical for phase separation. Second, post-lysis centrifugation retains only the soluble supernatant, likely eliminating key cellular elements such as membranes, cytoskeletal structures, and pre-existing condensates that actively contribute to condensate regulation in vivo. Finally, lysates lack the spatial compartmentalization of intact cells; nuclear and cytoplasmic components are homogenized, potentially altering condensate kinetics, composition, and material properties relative to their native subcellular environments.

2. The authors repeatedly describe their assay conditions as "near physiological." However, the experimental design relies on supplementing cell lysates with recombinant scaffold proteins (G3BP1 or NPM1). Based on the reported phase diagrams (Fig. 1h,i), the concentrations of added G3BP1 span ~3–40 μM, and NPM1 reaches up to ~100 μM. It is unclear how these concentrations compare with endogenous intracellular levels. However, G3BP1 in cells cannot spontaneously form condensates and requires induction by various stress conditions. This suggests that the lysate environment differs from that in intact cells—could the authors provide some discussion on this discrepancy?
If the supplemented scaffold proteins substantially exceed physiological abundance, they may dominate the phase behavior and obscure the regulatory contributions of more subtle lysate components. The authors should therefore quantify the endogenous concentrations of G3BP1 and NPM1 in the lysate and report the corresponding fold increase introduced by recombinant supplementation. Such information is essential for evaluating the extent to which the observed phase behavior reflects physiologically relevant regulation versus effects driven primarily by excess scaffold protein.
Does varying the concentration of cell lysate reflect conditions that are physiologically meaningful? According to BioNumbers, the average cellular density is ~1.04 g cm^{-3} (https://bionumbers.hms.harvard.edu/bionumber. aspx?id=110792&ver=1&trm=cell+density&org=), with relatively limited variation across cell types. Although local macromolecular concentrations can vary within subcellular compartments, the bulk cellular concentration is expected to remain close to this value. In contrast, the authors explore an exceptionally broad range of lysate concentrations (0–2 mg mL^{-1} for G3BP1 lysate and 0–9 mg mL^{-1} for NPM1 lysate; Fig. 1), conditions that are unlikely to be encountered in intact cells. It would therefore be important for the authors to clarify the rationale underlying the choice of this parameter space and to discuss how these concentrations relate to, or approximate, physiologically relevant intracellular environments.
The lysate preparation includes 0.5% NP40. Although this is a standard procedure, nonionic detergents are known to perturb hydrophobic interactions—an effect particularly relevant given that the authors later probe hydrophobic contributions to phase separation using 1,6-hexanediol. It would therefore be important to discuss whether residual NP40 may influence intermolecular hydrophobic interactions and consequently shift the observed phase boundaries. If technically feasible, the authors should evaluate whether removal or reduction of the detergent alters the phase diagrams. If such experiments are not possible, this limitation should be explicitly acknowledged and its potential impact on the interpretation of the results discussed.
Lysate-based systems are inherently susceptible to batch-to-batch variability. Although Supplementary Fig. 1f demonstrates reproducibility for NPM1, the manuscript provides limited information on how lysate preparation and normalization were controlled. The authors should clarify in the Methods or Results how lysates were standardized across experiments. Specifically, were lysates normalized solely by total protein concentration, or were endogenous levels of the relevant scaffold proteins also quantified to ensure consistency across batches? Explicitly addressing these points would improve confidence in the robustness and reproducibility of the platform.

3. Although the authors emphasize that ExVivo PhaseScan operates under more physiologically relevant conditions, no data are presented to demonstrate that the molecular composition or relative abundances of components within ExVivo PhaseScan–derived condensates are consistent with those measured in vivo. Moreover, nucleoli in cells are known to exhibit a multiphase, subcompartmentalized organization. It remains unclear whether such structural features are recapitulated in the nucleolar condensates generated using ExVivo PhaseScan. Providing compositional validation or evidence of multiphase organization would substantially strengthen the claim of physiological relevance.

4. Figure 3 convincingly demonstrates that the ExVivo system recruit's client proteins such as FUS and IL-38 more efficiently than the recombinant system. However, to establish that this recruitment is specific rather than arising from nonspecific crowding or promiscuous interactions within the lysate environment, it would be important to test proteins not expected to partition into stress granules. Inclusion of such negative controls would further substantiate the specificity of client recruitment and strengthen the reliability of the platform as a screening tool.

5. The authors developed a machine learning algorithm to classify condensate morphology. Was the data used to train the

algorithm derived from ExVivo PhaseScan experiments or recombinant condensate experiments? How many images were used for training? What are the quantitative indicators for distinguishing aggregates from condensates? These details should be fully described in the Supplementary Information methods.

6. The authors developed a machine-learning–based classifier (MobileNetV2 coupled with XGBoost) to distinguish homogeneous, condensate, and aggregate states. Although the confusion matrix indicates high classification accuracy, the model appears to rely primarily on morphological features. While the insets in Fig. 4 present FRAP measurements, these seem to be shown only for representative conditions. It remains unclear whether a broad population of droplets classified as “Aggregates” consistently exhibits the expected lack of fluidity in FRAP assays. Moreover, does the presence of an “irregular internal structure” correlate unambiguously with a solid or arrested state? Providing quantitative FRAP recovery data across a larger set of droplets and directly correlating recovery kinetics with morphological classes would substantially strengthen the conclusions.
The manuscript assesses condensate material properties using FRAP and classifies morphological states via machine-learning approaches under multiple conditions. However, biomolecular condensates—particularly those formed by ALS-associated mutants—are known to undergo time-dependent maturation or liquid-to-solid transitions. The authors should therefore explicitly report the reaction time, or on-chip residence time, prior to imaging. In addition, providing data or appropriate control experiments demonstrating that the measured material properties (e.g., FRAP recovery kinetics) and morphological classifications remain stable, or are at least well characterized, over the relevant time window would strengthen the interpretation of the results and clarify potential aging effects.

7. In line 237, the authors compare ExVivo PhaseScan with recombinant condensates. Is the RNA concentration used for recombinant condensates reasonable? Is it supported by the literature, or does this concentration possess specific characteristics? Furthermore, recombinant condensates lack the crowded environment present in cell lysates—would the results remain consistent after supplementing with crowding agents such as PEG or dextran?

8. The authors aim to observe the recruitment of WT FUS and FUS mutants to stress granules formed in ExVivo PhaseScan. I am curious why it is not feasible to directly induce stress granules with drugs in the lysate and observe the recruitment of different FUS variants directly?

9. For the experiments described in line 273 (NPM1 with the G156E lysate and stress granules using G3BP1 with the R522G lysate), could a colocalization experiment be performed to verify that FUS is indeed recruited into NPM1 and G3BP1 condensates, rather than forming other types of condensates?

10. The authors identified an RNA aptamer that can alter the quantitative phase diagram involving FUS mutants and prevent the formation of aggregates. Could the authors supplement with a cellular experiment by transfecting this RNA aptamer into cells to observe changes in the dynamic behavior of FUS mutants in stress granules and nucleoli?

Minor points:

1. What is the meaning of "scaffold dominance" mentioned in line 181? Readers may not have an intuitive understanding, and a clear explanation should be provided in the main text.

2. "Phase landscape" and "recruitment landscape" mentioned in lines 249 and 251 should also be clearly defined in the main text to clarify their specific meanings.

3. In Fig. 4b, the same image appears twice during algorithm training and validation. Does the repeated image have any specific significance? This could easily mislead readers into thinking that the training set and test set are the same dataset.

(Remarks on code availability)

Reviewer #4

(Remarks to the Author)
I co-reviewed this manuscript with one of the reviewers who provided the listed reports. This is part of the Nature Communications initiative to facilitate training in peer review and to provide appropriate recognition for Early Career Researchers who co-review manuscripts.

(Remarks on code availability)

Version 1:

Reviewer comments:

Reviewer #1

(Remarks to the Author)
This manuscript presents a new approach for studying protein condensates in a lysate based platform. This approach, the authors claim that retains substantially greater biochemical complexity than reported systems till date. I feel, the approach still falls short of undersatnding the intracellular environment. Physiologically, a condensate is regulated by a multitude of factors, like signaling pathways, spatial organization, posttranslational modifications, and other regulatory mechanisms. Hence, the title of the paper itself is not justified "Mapping high resolution, multidimensional phase diagrams of physiological
1
protein condensates". Also, lysate preparation inherently involves biochemical extraction, detergent interference and dilution steps that disrupt native compartmentalization and may alter the abundance, native folding or activity of endogenous cofactors.
I agree with the authors view that the system as being more physiologically relevant than existing in vitro approaches. The experiments conducted, primarily focuses on protein constituents and their effect on condensate formation. Physiological condensates are composed of many different types of biomolecules, including nucleic acids, lipids, metabolites, and organelle associated components. The manuscript does not sufficiently address the relative contribution of these non-protein constituents, nor does it provide detailed analyses of how closely their abundance and organization resemble those found in living cells. Here a simple detection of the components is not sufficient to establish physiological relevance.
For example, it remains unclear what fraction of the condensate mass or volume is occupied by proteins relative to other biomolecular constituents. If the authors wish to claim that their system more closely mimic physiological condensates, a more comprehensive characterization of the non-protein components and their functional contributions would be necessary.
The authors also provide an over interpretation of their data by concluding that low-affinity hydrophobic contacts, protein RNA-mediated bridging, complementary charge patters are present in the interactions. There are feeble experimental evidences (experiments with 1,2-hexanediol or KCl). However, I donot see any convincing experiment that would enable us to understand which of these interactions dominate under which condition. I appreciate the plethora of experiments conducted in the entire manuscript. However, the way the results are interpreted does not add anything new to the existing knowledge.
I appreciate the technical advances of the manuscript in putting forward this platform as an intermediate experimental mofdel between simplified recombinant systems and living cells. However, I do not believe that the current study provides enough evidence to support the broader claims of physiological relevance required for publication in Nature Communications. Therefore, I am unable to recommend the manuscript for publication in its present form.

(Remarks on code availability)

Reviewer #2

(Remarks to the Author)
The authors have made considerable efforts to address my concerns and improve their manuscript. I now have no more concerns and feel that the manuscript is suitable for publication.

(Remarks on code availability)

Reviewer #3

(Remarks to the Author)
The authors have adequately responded to reviewer comments.

(Remarks on code availability)

Reviewer #4

(Remarks to the Author)
I co-reviewed this manuscript with one of the reviewers who provided the listed reports. This is part of the Nature Communications initiative to facilitate training in peer review and to provide appropriate recognition for Early Career Researchers who co-review manuscripts.

(Remarks on code availability)

Open Access This Peer Review File is licensed under a Creative Commons Attribution 4.0 International License, which permits use, sharing, adaptation, distribution and reproduction in any medium or format, as long as you give appropriate credit to the original author(s) and the source, provide a link to the Creative Commons license, and indicate if changes were made.
In cases where reviewers are anonymous, credit should be given to 'Anonymous Referee' and the source.
The images or other third party material in this Peer Review File are included in the article's Creative Commons license, unless indicated otherwise in a credit line to the material. If material is not included in the article's Creative Commons license and your intended use is not permitted by statutory regulation or exceeds the permitted use, you will need to obtain permission directly from the copyright holder.
To view a copy of this license, visit https://creativecommons.org/licenses/by/4.0/

RESPONSE TO REVIEWERS' COMMENTS

Reviewer #1:

This manuscript presents a nice approach by using cell lysate as a biologically relevant crowding environment to study liquid-liquid phase separation (LLPS), moving beyond the use of synthetic crowders such as PEG/dextran in the in vitro studies. The overall concept is well motivated and has the potential to significantly advance the field by bringing in vitro LLPS studies closer to physiological conditions. However, in its current form, the manuscript requires several improvements to strengthen mechanistic insight, methodological clarity, and contextualization. I therefore recommend major revision before the work can be considered for publication.

We thank the Reviewer for their time, enthusiasm and constructive comments and have addressed their outstanding concerns as detailed below.

1. The central novelty of the study lies in the use of cell lysate, which inherently contains a complex mixture of proteins, lipids, nucleic acids, and other biomolecules. Despite this, if I understand correctly, the lysate concentration is largely treated as equivalent to protein concentration throughout the manuscript. While proteins may represent the dominant component, other biomolecules similar to the crowders used in the in vitro systems are known to play important roles in LLPS and may significantly influence the observed phase behaviour. This simplifying assumption limits mechanistic interpretation and underutilizes the key strength of the experimental design.

In the original manuscript, lysate concentration was reported using total protein concentration as a practical normalisation metric, but this may have implied that protein concentration alone was being treated as the relevant determinant of phase behaviour. This was not our intention. In ExVivo PhaseScan, the "lysate concentration" axis represents a dilution series of the complete soluble lysate fraction, normalised by total protein concentration. Thus, as lysate concentration is varied, retained lysate-derived proteins, RNAs, lipids, metabolites and other soluble components are co-varied. We therefore do not interpret the lysate axis as a protein-only variable, but as a tractable measure of total lysate input. We have now clarified this point in the revised manuscript (lines 146-150). In addition, to better understand the molecular complexity and comparability of the lysate samples, we have now added compositional analyses of the lysate, including RNA-seq, proteomics and TLC-based lipid profiling, as described below in response to point 2. We also have now benchmarked RNA, protein and lipid abundance across the various lysates used in the study, as described in response to point 3. These additions now make explicit that lysate-derived non-protein components may well contribute to condensation, while also standardising lysate inputs across experiments. We thank the reviewer for this useful point of clarification.

2. A more systematic characterization of the lysate composition is therefore necessary. At present, the absence of detailed compositional analysis makes it difficult to assess the relative contributions of different biomolecular classes. Even a coarse or semiquantitative analysis of major components, at minimum proteins, nucleic acids, lipids, and relevant inorganic salts, would provide essential context and greatly improve the depth and rigor of the study. This is important as in different physiological conditions, these molecules may present a different concentration profile.

To provide a compositional characterisation of the lysate, we have now performed RNA-seq (Table 2; Supplementary Fig. 1b), proteomics (Table 3; Supplementary Fig. 1c), and thin-layer chromatography (TLC) to assess major lipid classes (Supplementary Fig. 1d). Transcriptomic and proteomic enrichment analyses revealed strong representation of cytoplasmic and organelle-associated components, while TLC profiling identified a heterogeneous mixture of major lipid classes, including phosphatidylserine

(PS), phosphatidylcholine (PC), phosphatidylethanolamine (PE), and additional lipid species. Because all lysates were prepared and diluted in the same defined buffer system, the inorganic salt contribution from the lysates is negligible, and is instead determined by the buffers.

3. The rationale behind the concentration dependence of the lysate also requires clearer explanation. The authors should explicitly discuss what physicochemical changes are expected as lysate concentration is varied and why protein concentration alone is used as the defining parameter. Clarifying this point would significantly improve transparency in experimental design and data interpretation. In this context, it would also strengthen the manuscript to include a discussion of potential differences between lysates derived from normal versus diseased cells. While experimental validation may be challenging, even a conceptual or simulation based discussion of disease associated changes in lysate composition and their possible consequences for LLPS would broaden the relevance of the work.

We agree that this required clearer explanation. As outlined in response to point 1, total protein concentration is used as a practical normalisation metric for lysate loading, not as an indication that protein alone determines phase behaviour. We have now clarified in the manuscript that varying lysate concentration co-varies the retained soluble lysate components, including proteins, RNAs, lipids, metabolites and cofactors (lines 146-150). This is expected to alter macromolecular crowding, RNA/protein stoichiometry and client/cofactor availability.

To address whether differences in starting lysate composition could confound comparisons between lysates (beyond modulation of the desired client), we performed additional coarse-grained compositional analyses across lysates. Specifically, we measured RNA, protein and lipid abundance across G3BP1-mScarlet, NPM1-mScarlet and FUS-mEmerald WT, G156E and R522G lysates. RNA concentrations were measured by NanoDrop, protein concentrations by BCA assay, and broad lipid classes by TLC profiling. These measurements showed that the lysates were very similar across the measured parameters (Supplementary Fig. 7b). We also confirmed previously that FUS WT, G156E and R522G lysates had comparable FUS reporter expression levels (Supplementary Fig. 7a).

We also agree that lysates derived from different physiological or disease states will very likely differ in specific proteins, RNAs, lipids or metabolites, and that these differences could shift phase boundaries, material properties or client recruitment. We have added this point to the Discussion as a future potential application of ExVivo PhaseScan (line 454-458). In our revised manuscript, the new compositional analyses provide a coarse benchmark showing that the lysates compared here are broadly similar at the level of total RNA, protein and lipid abundance, but we acknowledge that specific molecular differences may still contribute to condensate behaviour.

4. The manuscript does not compare with existing in vitro LLPS studies involving the proteins of interest, particularly those conducted using defined crowding agents. Such a comparison is important to clearly understand what new insights are placed forward by the lysate based approach and to properly position the study within the existing LLPS literature.

As the reviewer notes, many recombinant G3BP1 LLPS behaviours have been characterised in detail using purified components and defined crowding agents, so rather than repeating this literature, we added a targeted benchmark experiment. To achieve this, we performed additional PhaseScan experiments using recombinant G3BP1 with PEG 10 kDa as a defined crowding agent, and titrated poly(A) RNA to compare our lysate-based system against a simplified recombinant system (Supplementary Fig. 4). Consistent with previous studies (PMID: 33597515), recombinant G3BP1-PEG condensates showed RNA-dependent condensation and re-entrant dissolution at higher RNA concentrations. Our ExVivo PhaseScan experiments showed a similar biphasic RNA dependence in

lysate-reconstituted stress granules (Fig. 2b-d), indicating that this behaviour is retained in a more compositionally complex environment. The key distinction though is that ExVivo PhaseScan allows this behaviour to be mapped while preserving lysate-derived proteins, RNAs, lipids and cofactors that are absent from the defined PEG system, enabling simultaneous analysis of phase boundaries, material properties and client recruitment. We have now also expanded the relevant Results (lines 216-220) and Discussion (lines 392-399) sections to better place our findings in the context of prior recombinant G3BP1 studies and highlight the novelties provided by this approach.

5. Several methodological details require clarification. In particular, the preparation of recombinant only condensates is insufficiently described, limiting reproducibility. Additionally, Figure 1a shows recombinant only condensates with predominantly circular morphology, whereas Figure 3b shows both recombinant only and lysate based condensates with irregular shapes. The authors should clarify whether this discrepancy arises from differences in experimental conditions, imaging parameters, or underlying material properties.

We have added an additional section to Methods section 7 describing the preparation of recombinant-only condensates (lines 709-716). The specific concentrations for each experiment are also listed in the corresponding figure legends. The morphological difference between Figure 1a and Figure 3b reflects differences in experimental conditions. The recombinant-only condensates shown in Figure 1a were formed with PEG, whereas the condensates in Figure 3b were formed with poly(A) RNA. In general, we find that condensates formed with crowding agents tend to wet the coverslip less than those formed in RNA/ lysate-based systems. This is likely because PEG promotes condensation largely through excluded-volume effects, whereas RNA-containing condensates are stabilised by multivalent interactions that alter interfacial properties and thus substrate interactions. As a result, RNA-containing or lysate-based condensates tend to partially wet or spread on the glass surface, producing less circular morphologies.

6. To substantiate the claim that lysate based condensates more closely resemble cellular condensates, direct and quantitative comparisons are needed. Specifically, material properties of cellular, lysate based, and recombinant only condensates may be compared using FRAP analysis. Without such comparisons, the claims are qualitative.

This is an important point. We have now performed direct FRAP comparisons between cellular stress granules, lysate-reconstituted stress granules, and recombinant G3BP1 condensates. Reconstituted stress granules displayed rapid fluorescence recovery on a timescale of seconds (Supplementary Fig. 2a), closely matching the recovery kinetics observed for stress granules in cells (Supplementary Fig. 2b). In contrast, recombinant condensates formed from G3BP1 with either poly(A) RNA (Supplementary Fig. 2c) or PEG (Supplementary Fig. 2d) showed slower recovery over the same timescale. These data provide quantitative support for our conclusion that lysate-reconstituted stress granules more closely resemble cellular stress granules than the simplified recombinant condensates tested here, at least with respect to their material properties. We have revised the manuscript to clarify this point (lines 128-136).

7. The interpretation of client partitioning data in Figure 3b requires some attention. First, client partitioning depends on both condensate material properties and scaffold client interactions. When the scaffold and nominal phase appear similar, what accounts for the differential partitioning of clients such as FUS, IL38, and PKM2? If the material phases of lysate based and recombinant only condensates differ, this should be clearly demonstrated and discussed before comparing partitioning behaviour using the same client molecules. Next, the lower fluorescence intensity observed for IL38 relative to FUS or PKM2 within condensates also requires explanation.

We agree that scaffold identity alone is insufficient to define equivalent condensate phases. Although the ‘lysate-based’ and ‘recombinant-only’ condensates both contain G3BP1 and are therefore nominally comparable as G3BP1-rich dense phases, they are compositionally and materially distinct. The recombinant-only condensates contain G3BP1 and poly(A) RNA, whereas the lysate-based condensates include a complex mixture of endogenous proteins, RNAs and lipids. These additional components can alter material properties and provide bridging interactions or binding sites that are absent from the minimal recombinant system. Consistent with this, our new FRAP analysis (discussed in response to point 6) shows that lysate-reconstituted stress granules recover on a timescale similar to cellular stress granules, whereas recombinant G3BP1 condensates formed with poly(A) RNA or PEG show slower recovery. The client-partitioning comparison is therefore not intended to compare identical material phases, but rather to test whether lysate-based reconstitution better captures cellular recruitment behaviour than a simplified recombinant G3BP1 condensate. In this context, the reduced recruitment of FUS, IL-38 and PKM2 into recombinant-only condensates likely reflects a combination of altered material properties and the absence of lysate-derived co-clients or bridging interactions, although the relative contribution of each mechanism is not distinguishable in this system. The lower apparent enrichment of IL-38 relative to FUS and PKM2 is explained by client-specific differences in partitioning, including differences in interaction valency, binding affinity, and overall compatibility with the dense-phase environment. We have now amended the results (lines 271-276) and discussion (lines 401-408) to clarify these points.

8. Also, the protein expression conditions raise concerns. The use of 500 mM IPTG for IL38 overexpression in E. coli BL21 (DE3) cells is unusually high and may promote protein aggregation. Is it a typo? The authors should clarify this choice and provide justification or evidence supporting proper protein folding and functionality under these conditions.

Thank you for spotting this typo. This should have read 500 µM. We have now corrected this.

The experimental concept is strong and more towards physiology of course, however, the current form of the manuscript does not yet justifies that. Addressing the above points would enhance the clarity and impact of the work and the conclusions of the study.

Reviewer #2:

Tanushree and colleagues present a lysate-based microfluidic platform aiming at generating high resolution phase diagram especially for physiological protein condensates. The platform, named ExVivo PhaseScan, was identical to their 2022 nature comm paper (called PhaseScan back then). The only difference I can tell is that they replace the in-vitro medium with cell lysates to mimic in-vivo conditions. So I think that technical advancement of this manuscript is minor. Application-wise, they described the lysate-reconstitution and applied the platform to dissect stress granule assembly and provide pathological insights. However, further evidence and much greater experimental rigor is needed to substantiate their claims and provide a truly novel advance in this field. Below are my concerns and comments

We thank the Reviewer for their time and careful assessment of our manuscript. We recognise the concern that ExVivo PhaseScan builds on the original PhaseScan microfluidic architecture. However, the central advance of our new study is not the invention of a new droplet generator, but the adaptation of this high-throughput phase-mapping framework to lysate-based reconstitution of multicomponent condensates. This enables phase boundaries, material states and client recruitment to be measured in a cell-free system that retains many endogenous proteins, RNAs, lipids and cofactors absent from simplified recombinant assays, while preserving the concentration control and scalability that are difficult to achieve in cells. We believe this represents a real step change towards studying condensates in more native states. In our revised manuscript we clarify this distinction more clearly and better define the specific advantages and limitations of ExVivo PhaseScan as discussed below.

Major:

1. Fig2: why not also barcoding concentrations of RNA/1,6-HEXANEDIOL/KCl using an additional dye color? In this way the screening would be in higher dimension and more comprehensive. It seems (in panel b) that these additives are in a broad range of concentrations rather than specific values. What's the point of doing that? did these concentrations also undergo arbitrary dilution through microfluidics? If so, I highly recommend barcode them as well. It is weird to put the data got from different RNA/1,6-HEXANEDIOL/KCl concentrations in the same plot. Or why not just do a serial dilution set of RNA/1,6-HEXANEDIOL/KCl concentrations instead of giving a range? It is at least clearer to see the differences if any.

The modulators in Figure 2 were indeed each barcoded with an Alexa Fluor dye (AF488), allowing their concentrations to be quantified in individual microdroplets. We agree that this was insufficiently clear in the original manuscript and have now clarified this in the Methods (lines 773-782) and relevant figure legends. These experiments were performed using microfluidic flow profiles that generate continuous dilution gradients of each modulator. The resulting concentration ranges are therefore not arbitrary and instead reflect the measured concentrations present in individual droplets after barcode calibration. This approach enables higher-dimensional phase mapping because scaffold, lysate and modulator concentrations can be quantified for each droplet. To make this clearer, we now include the full three-dimensional PhaseScan plots for the RNA (Figure 2b), 1,6-hexanediol (Figure 2g) and KCl (Figure 2i) experiments. Because three-dimensional plots can be difficult to interpret in a static figure, we have also retained two-dimensional concentration slices to show how the phase boundary changes across defined modulator concentration windows.

2. Lines 137–139: The claim that lysate-derived components are essential for condensation conflicts with the cannonical scaffold/client framework. Please clarify the specific role of these components in more detail.

We apologise that this was not clear in the original manuscript. We did not intend to imply that lysate-derived components replace G3BP1 or NPM1 as scaffolds, and thus that our data contradict the scaffold/client framework. Rather, our data show that, under the concentration and buffer conditions used here, recombinant G3BP1 or NPM1 alone does not undergo detectable condensation, whereas adding matched lysates to the corresponding recombinant scaffolds does induce condensation. Thus, G3BP1 and NPM1 remain the nucleating scaffold proteins in our assay, while lysate-derived components provide additional multivalent interactions, cofactors, clients or RNAs that support assembly of the reconstituted dense phase. We have revised the text to clarify that lysate-derived components are required for condensation in this specific experimental regime, rather than being defined as scaffolds themselves (lines 123-126; lines 162-167).

3. A key strength of this study is the use of lysates to introduce presumably physiological, endogenous components and native cofactors. However, the lysates were derived from cells overexpressing G3BP1/NPM1 and prepared with NP40 (containing detergent) and RNase inhibitors.
Furthermore, in the validation of client recruitment, the client proteins were also exogenously overexpressed, and precise modulation of biochemical variables was achieved by artificially adding exogenous mimics. Additionally, the identity and concentration of the native components retained in the lysates that could be recruited into the condensates were neither mentioned nor investigated.

These are important points; we have now performed additional controls (see below), revised the manuscript to clarify our rationale, and have added a new Limitations section to the Discussion (lines 440-449). Because NP-40 is a non-ionic detergent, it could in principle influence phase behaviour. To test this directly, we increased NP-40 above the concentration used in our lysis buffer, from 0.5% to 1% and 5%, and quantified condensate formation. NP-40 did not measurably alter lysate-reconstituted condensate formation under these conditions, even at 5%, which is tenfold higher than the concentration used for lysate preparation (Supplementary Fig. 2j).

With respect to exogenous expression and added modulators, our goal was to build a controllable cell-free system rather than to fully recreate the intracellular state. Stable expression of fluorescent G3BP1, NPM1 or FUS allowed us to track specific condensate markers or clients in lysates. For the client recruitment experiments, we used fluorescently tagged client reporters because suitable antibody-based detection was limited by antibody availability and by the potential for antibody binding to alter client partitioning through steric effects or avidity. These reporters were used to compare recruitment across matched conditions, rather than to infer absolute endogenous client abundance. Poly(A) RNA, KCl and 1,6-hexanediol were used as defined perturbations to test the contribution of RNA-dependent, electrostatic and hydrophobic interactions. These physicochemical parameters are currently impossible to vary quantitatively and independently inside cells, illustrating an important advantage of ExVivo PhaseScan.

To better define the native components retained in the lysate, we have now added RNA-seq, proteomics and TLC-based lipid profiling (Table 2; Table 3; Supplementary Fig. 1b-d). Transcriptomic and proteomic enrichment analyses revealed strong representation of cytoplasmic and organelle-associated components, while TLC profiling identified a heterogeneous mixture of major lipid classes, including phosphatidylserine, phosphatidylcholine, phosphatidylethanolamine and additional lipid species. We also have now performed coarse-grained compositional comparisons across G3BP1-mScarlet, NPM1-mScarlet and FUS-mEmerald WT, G156E and R522G lysates, measuring total RNA, protein and lipid abundance (Supplementary Fig. 7b). These measurements showed similar lysate compositions across the measured parameters. Finally, we previously confirmed that FUS WT, G156E and R522G lysates had comparable FUS reporter expression levels (Supplementary Fig. 7a). We have amended the manuscript with these additional experiments and discussion.

4. The authors used the ExVivo PhaseScan platform to study stress granule assembly, variable modulation, and condensate maturation. While the observations align with the cited literature, novel findings that specifically demonstrate the platform's advantages over PhaseScan would better highlight its significance.

We agree that the advantages of ExVivo PhaseScan over the original PhaseScan platform needed to be stated more explicitly. The advance is not that lysate replaces a defined *in vitro* buffer. By combining lysate reconstitution with PhaseScan, ExVivo PhaseScan enables high-resolution phase mapping of condensates assembled in the presence of endogenous lysate-derived proteins, RNAs, lipids and other cellular components, while retaining quantitative control over scaffold concentration and defined perturbations. This enables analyses that were not accessible in the original PhaseScan format, including meaurements of: (i) the physicochemical modulation of physiological-like multicomponent condensates (Figure2); (ii) client recruitment into lysate-reconstituted condensates and; (iii) high-throughput mapping of disease-linked FUS phase behaviour, including the rescue of pathological aggregate states by RNA aptamers. We have revised the Discussion to make this distinction clearer (lines 392-399). In particular, we now emphasise that ExVivo PhaseScan extends PhaseScan from mapping simplified recombinant phase behaviour to interrogating multicomponent, lysate-derived condensate systems in which phase boundaries, material properties, client recruitment and pathological phase transitions can be analysed in parallel.

Minor:

1. Fig1: I'm wondering whether the addition of fluorescent dye molecules affects the phase separation behavior?

We have now tested this directly by varying the concentration of the barcoding dye while holding G3BP1 lysate concentration constant. The resulting phase behaviour was unchanged, indicating that the barcoding dye does not measurably influence condensation under the conditions used here (Supplementary Fig. 2i).

2. Abstract: it would be better to identify ALS and FUS at the very first place of using these abbreviations.

We have now defined amyotrophic lateral sclerosis (ALS) and Fused in Sarcoma (FUS) at first use in the Abstract.

3. Several claims of "for the first time" in the Discussion appear overstated based on the data presented.

We agree and have removed claims of "for the first time".

Reviewer #3:

Agarwal et al. present ExVivo PhaseScan, an innovative droplet-based microfluidic platform designed to bridge the gap between the biochemical complexity of cellular lysates and the high-throughput, parameter-controlled precision of in vitro microfluidics. Using this approach, the authors generate high-resolution phase diagrams for two major biomolecular condensates—stress granules and nucleoli—and systematically examine the roles of RNA concentration, hydrophobic interactions, and electrostatic forces in driving phase separation. A major strength of the study is the demonstration that this lysate-based system more faithfully recapitulates client protein recruitment than simplified recombinant reconstitution assays. In addition, the integration of a machine-learning–based image classifier enables quantitative analysis of condensate morphology, revealing that ALS-associated FUS mutations shift the phase landscape toward pathological solid or aggregated states. Importantly, the authors further show that this aberrant behavior can be partially reversed using a specific RNA aptamer.

Overall, the data are of high quality, and the resulting phase diagrams exhibit impressive resolution. The application of machine learning for automated morphological classification represents a notable methodological advance. However, despite these strengths, I do not consider the manuscript to meet the conceptual or mechanistic threshold for Nature Communications. More detailed concerns are outlined below.

We thank the Reviewer for their time and thoughtful assessment of our manuscript, and for recognising the quality of the data presented. We also appreciate the concern that the conceptual distinction from the original PhaseScan platform and the physiological interpretation of lysate-based reconstitution needed to be stated more precisely. In the revised manuscript, we have therefore clarified that ExVivo PhaseScan is not intended to fully recapitulate the intracellular environment. Rather, it provides a controlled, scalable lysate-reconstitution framework that preserves many endogenous lysate-derived components while enabling quantitative perturbations and high-resolution phase-boundary mapping that are not possible in intact cells.

Major points:

1. Quantitative characterization of phase diagrams for multicomponent biomolecular condensates under near-physiological conditions is essential for elucidating the molecular mechanisms governing condensate composition and regulation. However, conventional approaches face inherent limitations: in vitro reconstitution systems are often overly simplified, whereas in vivo cellular assays are difficult to control and systematically manipulate. To address this gap, Agarwal et al. introduce a microfluidic strategy in which cell lysates are incubated with scaffold proteins, enabling high-throughput sample preparation and real-time visualization of condensate formation. This approach allows rapid construction of quantitative phase diagrams for stress granules and nucleoli. Despite these technical advances, the conceptual distinction between the present study and the authors' 2022 work (https://www.nature.com/ articles/s41467-022-35265-7), appears limited, aside from replacing a defined component with cell lysate. While incorporating lysates is a reasonable attempt to enhance physiological relevance and potentially extend the PhaseScan platform toward drug-screening applications—an advance of clear interest to the biophysics, cell biology, and pharmacology communities—it raises a fundamental concern regarding how faithfully cell lysates recapitulate intracellular conditions. First, lysate preparation requires substantial buffer dilution and includes NP40, a detergent that may perturb intermolecular interactions critical for phase separation. Second, post-lysis centrifugation retains only the soluble supernatant, likely eliminating key cellular elements such as membranes, cytoskeletal structures, and pre-existing condensates that actively contribute to condensate regulation in vivo. Finally, lysates lack the spatial compartmentalization of

intact cells; nuclear and cytoplasmic components are homogenized, potentially altering condensate kinetics, composition, and material properties relative to their native subcellular environments.

We agree with the Reviewer that no reconstitution system can fully recapitulate the intracellular environment. This is, of course, an inherent limitation of not working in live cells, and we have added a dedicated Limitations section to the revised Discussion to state this explicitly. As such, ExVivo PhaseScan is not intended to replace live-cell experiments or to claim that lysate condensates are identical to their cellular counterparts. Rather, the platform is designed to occupy an intermediate regime between minimal recombinant reconstitution and intact cells. Compared with recombinant-only systems, which typically contain one or two components, lysate-based reconstitution retains a complex mixture of thousands of components, including endogenous proteins, RNAs, and lipids (see new data discussed in response to Point 2 below). Compared with cells, it allows defined physicochemical perturbations to be controlled quantitatively and screened at high throughput.

We also appreciate that lysate preparation introduces important caveats. Detergent exposure, dilution, centrifugation, and loss of nuclear/cytoplasmic compartmentalisation may all alter condensate composition, kinetics and material properties. We discuss this in our responses below and have also now highlighted these issues more clearly in the Limitations section in the Discussion (lines 440-449). We consider ExVivo PhaseScan as a reconstitution platform that enables the enrichment of native components, not as a complete mimic of the cellular interior. We address the specific concern regarding NP-40 below.

2. The authors repeatedly describe their assay conditions as "near physiological." However, the experimental design relies on supplementing cell lysates with recombinant scaffold proteins (G3BP1 or NPM1). Based on the reported phase diagrams (Fig. 1h,i), the concentrations of added G3BP1 span ~3–40 µM, and NPM1 reaches up to ~100 µM. It is unclear how these concentrations compare with endogenous intracellular levels. However, G3BP1 in cells cannot spontaneously form condensates and requires induction by various stress conditions. This suggests that the lysate environment differs from that in intact cells—could the authors provide some discussion on this discrepancy? If the supplemented scaffold proteins substantially exceed physiological abundance, they may dominate the phase behavior and obscure the regulatory contributions of more subtle lysate components. The authors should therefore quantify the endogenous concentrations of G3BP1 and NPM1 in the lysate and report the corresponding fold increase introduced by recombinant supplementation. Such information is essential for evaluating the extent to which the observed phase behavior reflects physiologically relevant regulation versus effects driven primarily by excess scaffold protein.

We agree that the concentrations of added scaffold protein should be interpreted carefully. Using cellular concentrations from the HeLa spatial proteome (PMID: 27278775), G3BP1 is present at approximately 2.0 µM and NPM1 at approximately 26.7 µM. Even though these values are within the same order-of-magnitude as the scaffold concentrations explored in ExVivo PhaseScan, the assay should not be interpreted as a direct recreation of endogenous intracellular scaffold concentrations. Rather, in both the G3BP1 and NPM1 systems, recombinant scaffold concentration is used as a controlled nucleation parameter that allows phase boundaries to be mapped quantitatively across a defined range of scaffold and lysate inputs.

This distinction relates to the Reviewer's second point because condensate formation in cells is not governed by scaffold concentration alone. Stress granule and nucleolar assembly are shaped by upstream signalling, RNA availability, post-translational regulation, local concentration effects, transport, and spatial organisation, many of which are altered or absent in cleared lysate. We have now added this discussion to the new Limitations section (lines 440-449) to state more explicitly that ExVivo PhaseScan does not aim to reproduce spontaneous intracellular condensate formation but

instead provides a controlled lysate-reconstitution framework for asking how scaffold availability, lysate-derived components and defined physicochemical perturbations shape condensate phase behaviour.

Does varying the concentration of cell lysate reflect conditions that are physiologically meaningful? According to BioNumbers, the average cellular density is ~1.04 g cm^{-3} (https://bionumbers.hms.harvard.edu/bionumber. aspx?id=110792&ver=1&trm=cell+density&org=) , with relatively limited variation across cell types. Although local macromolecular concentrations can vary within subcellular compartments, the bulk cellular concentration is expected to remain close to this value. In contrast, the authors explore an exceptionally broad range of lysate concentrations (0–2 mg mL^{-1} for G3BP1 lysate and 0–9 mg mL^{-1} for NPM1 lysate; Fig. 1), conditions that are unlikely to be encountered in intact cells. It would therefore be important for the authors to clarify the rationale underlying the choice of this parameter space and to discuss how these concentrations relate to, or approximate, physiologically relevant intracellular environments.

The cellular density value cited by the Reviewer reflects the bulk mass density of intact cells, which are predominantly water, and is therefore not directly comparable to the total soluble protein concentration used to normalise lysate input in ExVivo PhaseScan. In our assay, lysate concentration is reported as total protein concentration because this provides a practical and reproducible normalisation metric for the retained soluble lysate fraction. The lysate axis should therefore be interpreted as an operational dilution series of retained lysate components, rather than as a direct recreation of whole-cell mass density or intracellular macromolecular concentration. The purpose of varying lysate input over a broad range is to map phase boundaries and determine how increasing lysate-derived molecular complexity shifts condensation, not to claim that every point in the diagram corresponds to a physiological intracellular concentration. We apologise that this was not clear and have clarified this distinction in the revised manuscript (lines 146-150).

The lysate preparation includes 0.5% NP40. Although this is a standard procedure, nonionic detergents are known to perturb hydrophobic interactions—an effect particularly relevant given that the authors later probe hydrophobic contributions to phase separation using 1,6-hexanediol. It would therefore be important to discuss whether residual NP40 may influence intermolecular hydrophobic interactions and consequently shift the observed phase boundaries. If technically feasible, the authors should evaluate whether removal or reduction of the detergent alters the phase diagrams. If such experiments are not possible, this limitation should be explicitly acknowledged and its potential impact on the interpretation of the results discussed.

Because NP-40 is a non-ionic detergent, we agree that it could in principle perturb hydrophobic interactions and alter phase behaviour. To test this directly, we increased NP-40 above the concentration used in lysate preparation, from 0.5% to 1% and 5%, and quantified condensate size as a readout of condensate formation. NP-40 did not measurably alter lysate-reconstituted condensates under these conditions, even at 5%, which is tenfold higher than the concentration used for lysate preparation (Supplementary Fig. 2j).

Lysate-based systems are inherently susceptible to batch-to-batch variability. Although Supplementary Fig. 1f demonstrates reproducibility for NPM1, the manuscript provides limited information on how lysate preparation and normalization were controlled. The authors should clarify in the Methods or Results how lysates were standardized across experiments. Specifically, were lysates normalized solely by total protein concentration, or were endogenous levels of the relevant scaffold proteins also quantified to ensure consistency across batches? Explicitly addressing these points would improve confidence in the robustness and reproducibility of the platform.

Lysates were standardised by total protein concentration before use in ExVivo PhaseScan, and the same preparation and dilution workflow was used across experiments. We did not normalise lysates by endogenous G3BP1 or NPM1 abundance, because in this assay condensate nucleation is driven by the externally added recombinant scaffold, whose concentration is directly measured in each droplet by fluorescence barcoding. Thus, endogenous scaffold levels in the lysate are retained as part of the lysate background rather than used as the primary normalisation axis. To test whether gross differences in lysate composition could confound comparisons, we have now performed coarse-grained compositional analyses across the lysates used in the study, measuring total RNA, protein and lipid abundance in G3BP1-mScarlet, NPM1-mScarlet and FUS-mEmerald WT, G156E and R522G lysates. These measurements showed similar compositions across the measured parameters (Supplementary Fig. 7b). For the FUS mutant comparisons, we also confirmed previously that FUS WT, G156E and R522G reporter expression levels were comparable across lysates (Supplementary Fig. 7a). We have clarified in the Methods that lysates were standardised by total protein concentration and that recombinant scaffold concentration, rather than endogenous scaffold abundance, defines the controlled scaffold axis in ExVivo PhaseScan (lines 578-583).

3. Although the authors emphasize that ExVivo PhaseScan operates under more physiologically relevant conditions, no data are presented to demonstrate that the molecular composition or relative abundances of components within ExVivo PhaseScan–derived condensates are consistent with those measured in vivo. Moreover, nucleoli in cells are known to exhibit a multiphase, subcompartmentalized organization. It remains unclear whether such structural features are recapitulated in the nucleolar condensates generated using ExVivo PhaseScan. Providing compositional validation or evidence of multiphase organization would substantially strengthen the claim of physiological relevance.

It is important to distinguish between compositional analysis of the input lysate, which we now provide, and that of the reconstituted condensates, which was not performed in this study but has been comprehensively performed previously. For the former, we have now carried out RNA-seq (Table 2; Supplementary Fig. 1b), proteomics (Table 3; Supplementary Fig. 1c), and thin-layer chromatography (TLC) to assess major lipid classes (Supplementary Fig. 1d). Transcriptomic and proteomic enrichment analyses revealed strong representation of cytoplasmic and organelle-associated components, while TLC profiling identified a heterogeneous mixture of major lipid classes, including phosphatidylserine (PS), phosphatidylcholine (PC), phosphatidylethanolamine (PE), and additional lipid species. These analyses show that the input lysate contains a broad mixture of cellular proteins, RNAs and lipid classes.

The original lysate-reconstitution study from the Taylor lab (PMID: 33502444) performed proteomic and transcriptomic analyses of lysate-reconstituted G3BP1 and NPM1 condensates and showed that these assemblies recruit many components of cellular stress granules and nucleoli. Our study builds on this validated reconstitution approach by coupling it to high-throughput phase mapping, rather than re-establishing the full condensate proteome/transcriptome from sorted condensates. We have now expanded the text to better connect our approach to the original lysate-reconstitution study from the Taylor lab (lines 103-106).

4. Figure 3 convincingly demonstrates that the ExVivo system recruit's client proteins such as FUS and IL-38 more efficiently than the recombinant system. However, to establish that this recruitment is specific rather than arising from nonspecific crowding or promiscuous interactions within the lysate environment, it would be important to test proteins not expected to partition into stress granules. Inclusion of such negative controls would further substantiate the specificity of client recruitment and strengthen the reliability of the platform as a screening tool.

We agree that this is an important control. We have now included recombinant GFP as a negative client control. GFP is not expected to partition into stress granules and did not enrich in either lysate-reconstituted stress granules or recombinant-only G3BP1 condensates (Supplementary Fig. 6). This supports the conclusion that recruitment of FUS, IL-38 and PKM2 is not simply due to nonspecific crowding or promiscuous client accumulation in the dense phase.

5. The authors developed a machine learning algorithm to classify condensate morphology. Was the data used to train the algorithm derived from ExVivo PhaseScan experiments or recombinant condensate experiments? How many images were used for training? What are the quantitative indicators for distinguishing aggregates from condensates? These details should be fully described in the Supplementary Information methods.

The images used to train the classifier were subsampled from ExVivo PhaseScan experiments, not recombinant condensate experiments. Training labels were assigned manually based on morphology, using criteria informed by previously reported quantitative descriptors of condensate-to-aggregate transitions, including intensity, area, roundness, circularity and aspect ratio (PMID: 40730560). Homogeneous droplets were defined as droplets with diffuse signal and no internal puncta. Condensate-containing droplets were defined by the presence of rounded, droplet-like internal structures. Aggregate-containing droplets were defined by irregular, high-intensity, non-spherical structures with reduced circularity and more heterogeneous internal morphology. These examples were then used to train MobileNetV2/XGBoost classifiers to assign each droplet as homogeneous, condensate-containing, or aggregate-containing. The number of labelled examples used for training were as follows: NPM1 model 1, 3,824 aggregate, 2,481 condensate and 9,257 homogeneous examples; NPM1 model 2, 3,860 aggregate, 2,524 condensate and 9,257 homogeneous examples; and G3BP1 model 3, 2,748 aggregate, 2,725 condensate and 10,924 homogeneous examples. Classifier performance is reported using confusion matrices and precision, recall and F1 scores are listed in Supplementary Fig. 7e-g. We have now expanded the Supplementary Text Section 2.1 to clarify the source of the training images, the number of labelled examples, and the morphological criteria used to define each class (lines 163-176) and cited this in the Methods section (lines 809-811) in the main article.

6. The authors developed a machine-learning–based classifier (MobileNetV2 coupled with XGBoost) to distinguish homogeneous, condensate, and aggregate states. Although the confusion matrix indicates high classification accuracy, the model appears to rely primarily on morphological features. While the insets in Fig. 4 present FRAP measurements, these seem to be shown only for representative conditions. It remains unclear whether a broad population of droplets classified as “Aggregates” consistently exhibits the expected lack of fluidity in FRAP assays. Moreover, does the presence of an “irregular internal structure” correlate unambiguously with a solid or arrested state? Providing quantitative FRAP recovery data across a larger set of droplets and directly correlating recovery kinetics with morphological classes would substantially strengthen the conclusions.

The machine-learning classifier provides a scalable morphological classification of droplets, whereas FRAP provides an orthogonal material-property validation for representative regions of the phase diagrams. The FRAP experiments shown in Figure 4 were collected from 3–4 independent experiments and are plotted as mean ± s.d. These measurements show rapid recovery for condensate-class assemblies and little or no recovery for aggregate-class assemblies in the sampled regions of the phase diagrams (Figure 4c,d,f,g inset panels). We include here for the Reviewer a quantitation of the % recovery at 7.5 seconds from each of the curves displayed in Figure 4. We agree that directly correlating FRAP recovery with morphology across a much larger droplet population would further strengthen this point. However, FRAP is intrinsically low throughput and cannot currently be applied at scale to the hundreds of thousands of droplets analysed by ExVivo PhaseScan. We therefore use

morphology as the scalable classification readout, supported by FRAP validation of representative condensate- and aggregate-class regions, rather than claiming that every aggregate-class droplet has been directly measured by FRAP. Supplementary Text Section 2.1 now details our rationale for the morphological criteria used to define each class and justify our approach (lines 163-176).

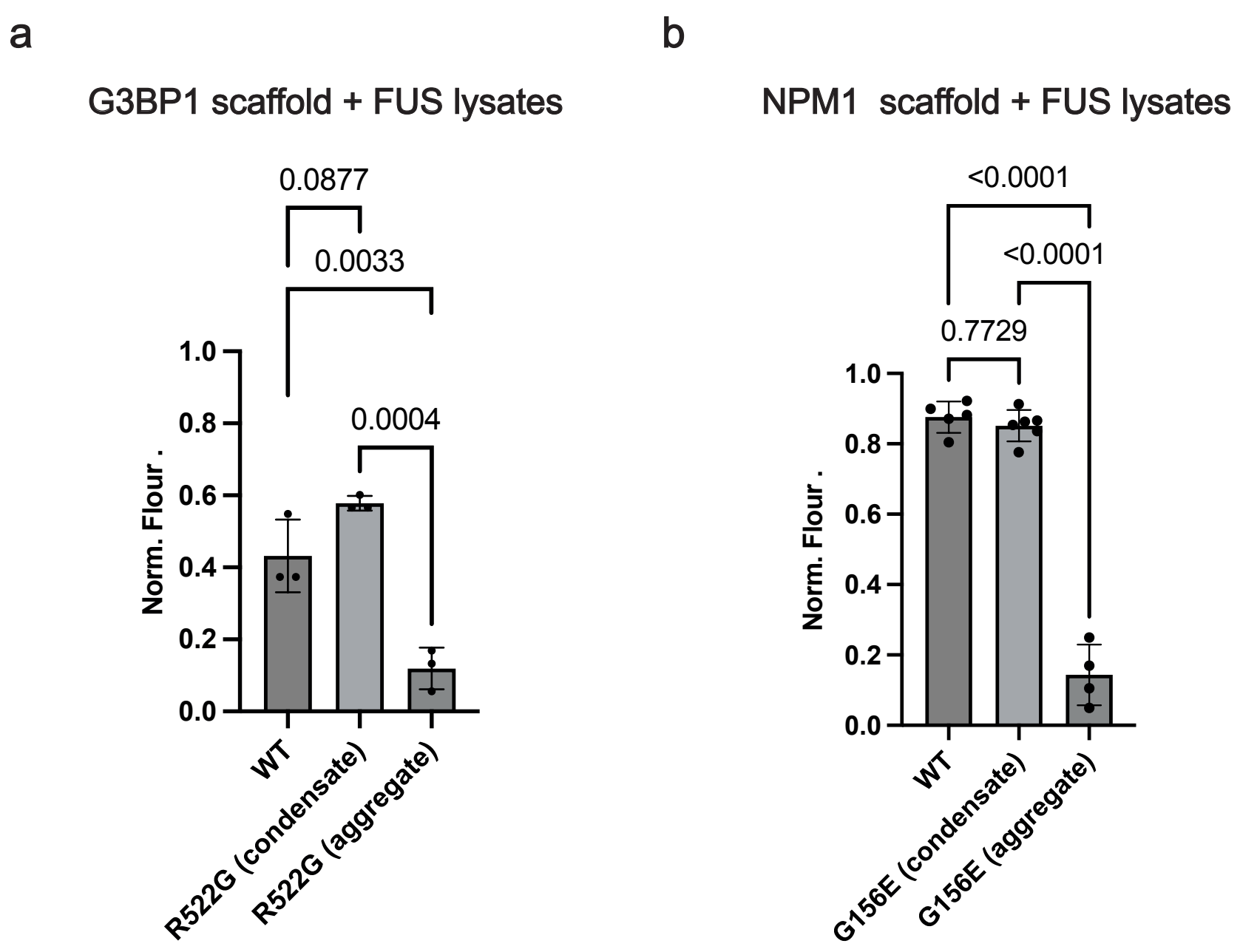

Reviewer Figure 1: Quantification of condensate/aggregate FRAP recovery. The figure displays FRAP recovery rates at 7.5 seconds after bleaching from: **(a)** 12.5 µM G3BP1 scaffold with 5 mg/mL FUS WT and 0.5 mg/mL (condensate), 5mg/mL (aggregate) R522G lysates **(b)** 45 µM NPM1 scaffold with 4 mg/mL FUS WT and 1.5 mg/mL (condensate), 4 mg/mL (aggregate) G156E lysates. Mean ± s.d, *n* = 3 (a); 4-6 (b) condensates/aggregates from independent experiments, One-way ANOVA with Tukey's multiple comparisons test.

The manuscript assesses condensate material properties using FRAP and classifies morphological states via machine-learning approaches under multiple conditions. However, biomolecular condensates—particularly those formed by ALS-associated mutants—are known to undergo time-dependent maturation or liquid-to-solid transitions. The authors should therefore explicitly report the reaction time, or on-chip residence time, prior to imaging. In addition, providing data or appropriate control experiments demonstrating that the measured material properties (e.g., FRAP recovery kinetics) and morphological classifications remain stable, or are at least well characterized, over the relevant time window would strengthen the interpretation of the results and clarify potential aging effects.

As the Reviewer mentions, condensate material properties change with time. A major advantage of the microfluidic setup is that reaction timing is tightly controlled: aqueous phases mix at the chip entry point and droplets are imaged after a defined on-chip residence time (345 seconds). Under the flow rates used here, droplets were imaged within a narrow temporal window that varied by less than 3 s between conditions. Thus, comparisons within a given PhaseScan experiment are made at effectively matched reaction times. We have now added the on-chip residence time and timing variability to the Methods (lines 791-793).

7. In line 237, the authors compare ExVivo PhaseScan with recombinant condensates. Is the RNA concentration used for recombinant condensates reasonable? Is it supported by the literature, or does this concentration possess specific characteristics? Furthermore, recombinant condensates lack the

crowded environment present in cell lysates—would the results remain consistent after supplementing with crowding agents such as PEG or dextran?

The RNA concentration used for the recombinant G3BP1 comparison was chosen based on prior recombinant G3BP1 LLPS studies (PMID:32302571). We have now cited this study in our revised manuscript (line 217). It would be interesting in future studies to compare how simultaneously varying RNA concentrations and crowding influenced client recruitment of recombinant condensates, however we see this as outside of the scope of the current manuscript.

8. The authors aim to observe the recruitment of WT FUS and FUS mutants to stress granules formed in ExVivo PhaseScan. I am curious why it is not feasible to directly induce stress granules with drugs in the lysate and observe the recruitment of different FUS variants directly?

Direct induction of stress granules in lysate is unlikely to reproduce cellular stress-granule assembly because most stress paradigms act through upstream signalling and translational control pathways that require intact cells. For example, sodium arsenite induces stress granules through activation of the integrated stress response, eIF2α phosphorylation and translational repression. These coordinated processes are not preserved in cleared lysate. Consistent with this, sodium arsenite does not efficiently induce stress granule formation in lysate-based systems. We therefore followed the strategy used in previous lysate-reconstitution work (PMID: 33502444), in which a small amount of recombinant scaffold protein is added to seed granule formation in a controlled manner.

9. For the experiments described in line 273 (NPM1 with the G156E lysate and stress granules using G3BP1 with the R522G lysate), could a colocalization experiment be performed to verify that FUS is indeed recruited into NPM1 and G3BP1 condensates, rather than forming other types of condensates?

We agree that this is an important control. We have now verified that FUS is recruited to the corresponding scaffold-positive assemblies in both systems. Specifically, FUS co-localises with NPM1-positive reconstituted nucleolar assemblies in the G156E lysate condition (Supplementary Fig. 7c) and with G3BP1-positive reconstituted stress-granule assemblies in the R522G lysate condition (Supplementary Fig. 7d). This supports the conclusion that FUS is recruited into the intended NPM1- or G3BP1-containing condensates/aggregates rather than forming independent assemblies.

10. The authors identified an RNA aptamer that can alter the quantitative phase diagram involving FUS mutants and prevent the formation of aggregates. Could the authors supplement with a cellular experiment by transfecting this RNA aptamer into cells to observe changes in the dynamic behavior of FUS mutants in stress granules and nucleoli?

We agree that testing the aptamer in cells would be a very interesting future direction. However, this experiment is unfortunately not straightforward. Transient delivery of unmodified RNA aptamers into cells by transfection is limited by cellular uptake and rapid nuclease-mediated degradation. Genetic expression is also non-trivial because short structured RNAs generally require a dedicated Pol III or engineered expression strategy, and expression level, processing and localisation would need to be validated before FUS condensate dynamics could be interpreted. Unfortunately, these optimisation steps are beyond the scope of what was achievable in the present revision timetable but we agree would be a very exciting future experiment. Instead, we have been careful not to claim that the aptamer result demonstrates cellular rescue. Instead, we present it as a proof-of-principle showing that ExVivo PhaseScan can identify RNA-based modulators that shift disease-linked FUS phase behaviour in a lysate-reconstituted system and provides a powerful tool for future screening efforts. We have revised the Discussion to clarify this point and to frame cellular delivery and validation of aptamer-based modulators as an important future direction (lines 433-439).

Minor points:

1. What is the meaning of "scaffold dominance" mentioned in line 181? Readers may not have an intuitive understanding, and a clear explanation should be provided in the main text.

Scaffold dominance (D_i) measures the fractional free energy contribution of species i to the total free energy of demixing. We have now added this into the main text.

2. "Phase landscape" and "recruitment landscape" mentioned in lines 249 and 251 should also be clearly defined in the main text to clarify their specific meanings.

We have now amended this to simply "…FUS recruitment…" and "…phase behaviour…"

3. In Fig. 4b, the same image appears twice during algorithm training and validation. Does the repeated image have any specific significance? This could easily mislead readers into thinking that the training set and test set are the same dataset.

The use of the same images in both parts of the figure was intended to illustrate how droplets were sorted into categories, which occurs downstream of training. However, to avoid any confusion, we have now changed these images.

Reviewer #4:

I co-reviewed this manuscript with one of the reviewers who provided the listed reports. This is part of the Nature Communications initiative to facilitate training in peer review and to provide appropriate recognition for Early Career Researchers who co-review manuscripts.
